# Motif Editing Reveals Hidden Active Sites in Atomically Precise Metal Nanoclusters for Enhanced Electrocatalysis

**DOI:** 10.1021/jacs.5c08684

**Published:** 2025-10-23

**Authors:** Zhihe Liu, Moshuqi Zhu, Bo Li, Junmei Chen, Shibo Xi, Yang-Yang Yu, Lu Xia, Lei Wang, De-en Jiang, F. Pelayo García de Arquer, Jianping Xie

**Affiliations:** a Department of Chemical and Biomolecular Engineering, 37580 National University of Singapore, 117585, Singapore; b School of Chemical Engineering and Technology, 12605 Tianjin University, Tianjin 300072, China; c ICFO − Institut de Ciències Fotòniques, The Barcelona Institute of Science and Technology, Castelldefels, Barcelona 08860, Spain; d Department of Chemical and Biomolecular Engineering, 5718 Vanderbilt University, Nashville, Tennessee 37212, United States; e Institute of Sustainability for Chemicals, Energy and Environment (ISCE2), Agency for Science, Technology and Research (ASTAR), 627833, Singapore; f Information Materials and Intelligent Sensing Laboratory of Anhui Province, Institutes of Physical Science and Information Technology, Anhui University, Hefei 230601, China; g Centre for Hydrogen Innovations, 37580 National University of Singapore, 1 Engineering Drive 3, Singapore 117585, Singapore; h Joint School of National University of Singapore and Tianjin University International Campus of Tianjin University Binhai New City, Fuzhou 350207, P. R. China

## Abstract

Metal nanoclusters offer atomically precise platforms for catalysis but often require bulk molecular motifs to achieve cluster stability. Here, we assess how these motifs block access to active sites and quantify trade-offs between structural integrity and catalytic performance. Based on this, we designed a motif-by-motif surface editing strategy to expose catalytic sites with atomic precision while preserving the kernel integrity of the cluster. Using [Au_25_(*p*MBA)_18_]^−^ nanoclusters (*p*MBA = para-mercaptobenzoic acid) as a model system, we selectively replace sterically bulky Au_2_(*p*MBA)_3_ motifs with compact Cu-(*p*MBA)_3_ units, yielding [Au_13_Cu_4_(*p*MBA)_12_]^3–^ nanoclusters with a symmetric, open-surface architecture. *In situ* absorption and mass spectrometry reveals a stepwise motif exchange mechanism distinct from conventional coreduction or ligand displacement, which enables surface reconstruction without kernel distortion. The resulting clusters deliver a 180-fold enhancement in hydrogen evolution turnover frequency (18.8 s^–1^), compared to the parent [Au_25_(*p*MBA)_18_]^−^ (0.1 s^–1^), attributed to increased Au_3_ facet exposure and improved hydrogen binding, as suggested by spectroscopy and density functional theory. This work offers a generalizable route to programmable surface engineering in metal nanoclusters, contributing to advance in the longstanding paradox between atomic precision and catalytic accessibility.

## Graphical abstract

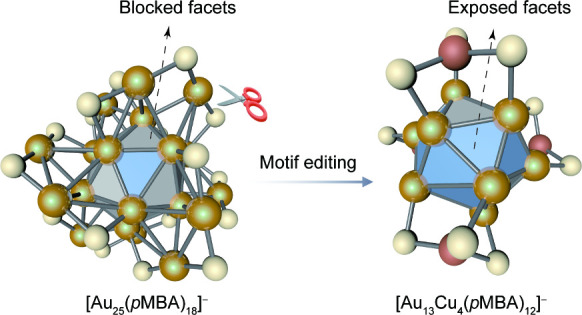

## Introduction

Advances in synthetic chemistry have positioned metal nanoparticles (NPs) as versatile platforms for electrocatalytic applications,^1,2^ due to their tunable high surface-to-volume ratios and abundance of under-coordinated surface atoms. However, achieving both high activity and stability remains a persistent challenge – smaller NPs, a priori more active, are inherently unstable and prone to aggregation unless stabilized by surface passivation.^3^ Besides shaping nanoparticle synthesis and electronic properties, organic ligands are commonly employed as stabilizers, protecting metal kernel.^4^ However, excessive ligand coverage often limits the number of exposed sites and further blocks access to other available sites, introducing trade-offs between net catalytic activity and stability.^5^

To mitigate this trade-off, surface modification strategies such as ligand removal or motif substitution have been widely explored.^6^ However, conventional approaches frequently induce disruptive structural rearrangements or alter kernel size, irreversibly degrading atomic-scale precision and catalytic efficacy.^7^ This highlights the need for strategies that precisely tailor surface accessibility without compromising kernel integrity.

Atomically precise metal nanoclusters (NCs, kernel size <3 nm) are attractive in this direction.^8–12^ With well-defined compositions and crystallographically resolved structures, metal NCs, particularly thiolate-protected gold clusters, provide an ideal model system for probing and customizing catalytic active sites at the atomic scale.^10,13–16^ These systems feature a metallic Au(0) kernel capped by Au­(I)-SR motifs, often in the form of Au_2_(SR)_3_ staples.^17–20^ However, such motifs extensively shield the kernel, with only a fraction of the catalytically active Au_3_ facets remaining reactant-accessible.^21,22^ While ligand exchange and motif displacement strategies have been explored, they often preserve steric hindrance or require harsh conditions, offering limited control over kernel exposure.^23–25^ Existing exchanges are typically limited to CN = 2-to-CN = 2 (CN = coordination number) substitutions and often yield statistical mixtures, rather than well-defined structural outcomes. As such, a general strategy for rational, kernel-preserving, atom-by-atom surface reconstruction remains elusive.

Here we present a motif editing strategy that enables atomically precise control over active-site exposure while fully preserving kernel geometry. Starting from [Au_25_(*p*MBA)_18_]^−^ model nanoclusters (*p*MBA = para-mercaptobenzoic acid), we selectively replace sterically bulky Au_2_(*p*MBA)_3_ motifs with compact Cu-(*p*MBA)_3_ units, leveraging the higher coordination preference of Cu­(I) to drive surface reconstruction. This yields [Au_13_Cu_4_(*p*MBA)_12_]^3–^ clusters with a symmetric structure and substantially reduced surface coverage, increasing the number of accessible Au_3_ facets from 2 to 16 (Figure 1a). We monitor these transformation pathways in real time via electrospray ionization mass spectra (ESI-MS), revealing a stepwise exchange mechanism distinct from conventional coreduction or ligand displacement. X-ray absorption spectroscopy and density functional theory (DFT) confirm that Cu atoms bind through thiolates without disturbing the Au_13_ kernel. This precise motif-by-motif editing simultaneously elevates coordination number and unblocks buried active sites, leading to a drastic improvement in catalytic performance: The resulting [Au_13_Cu_4_(*p*MBA)_12_]^3–^ NCs delivers a turnover frequency (TOF) of 18.8 s^–1^, 180-fold higher than [Au_25_(*p*MBA)_18_]^−^ (0.1 s^–1^) in hydrogen evolution reaction (HER). Our work proposes a rational and generalizable strategy to reconcile structural precision with functional accessibility in nanocluster electrocatalysis.

**Figure 1:**
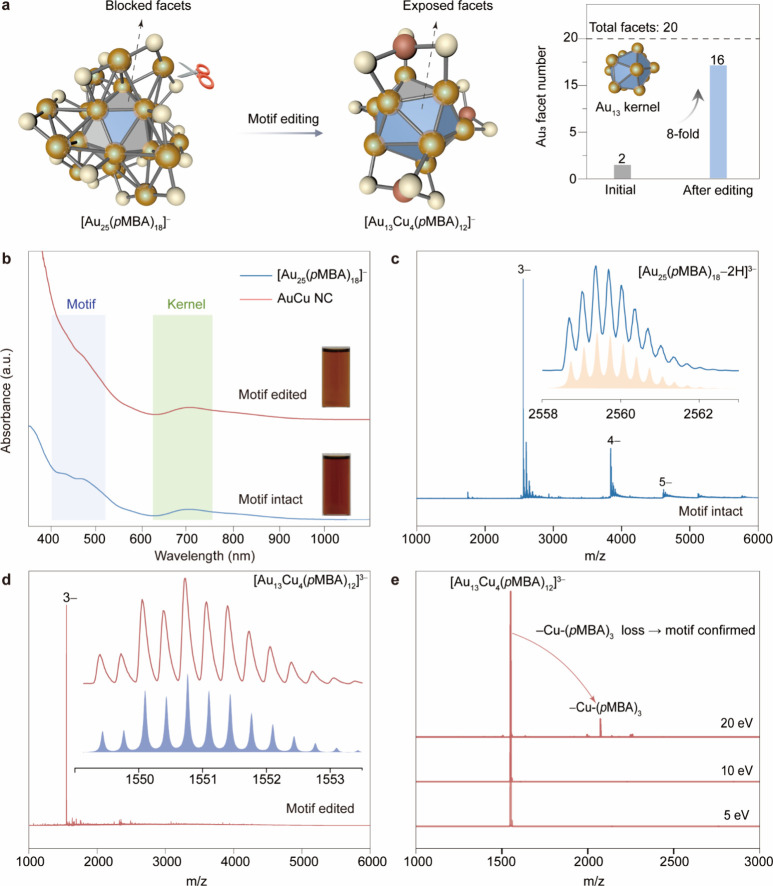
Structural characterization of motif-edited Au nanoclusters with kernel retention and facet exposure. (a) Schematic illustration of the surface motif editing strategy, transforming [Au_25_(*p*MBA)_18_]^−^ into [Au_13_Cu_4_(*p*MBA)_12_]^3–^ via rational substitution of Au_2_(*p*MBA)_3_ motifs by compact Cu-(*p*MBA)_3_ units, thereby exposing previously blocked Au_3_ facets on the icosahedral kernel. Right: the number of solvent-accessible Au_3_ facets increases from 2 to 16 (out of 20 total). (b) Ultraviolet–visible absorption spectra comparing [Au_25_(*p*MBA)_18_]^−^ (blue) into [Au_13_Cu_4_(*p*MBA)_12_]^3–^ (red). Insets: photographs of aqueous cluster solutions. (c) ESI-MS of [Au_25_(*p*MBA)_18_]^−^ displaying intact cluster signals (*e.g.*, [M–2H]^3–^), with inset showing agreement between simulated and experimental isotopic distributions. (d) ESI-MS of [Au_13_Cu_4_(*p*MBA)_12_]^3–^ with a dominant peak at *m*/*z* = 1550.9 and a matching simulated isotope pattern (inset), confirming its formula. (e) Tandem MS of [Au_13_Cu_4_(*p*MBA)_12_]^3–^ reveals a fragmentation product corresponding to loss of a Cu-(*p*MBA)_3_ unit under 20 eV collision energy, validating the presence of discrete surface motifs.

## Results and Discussion

We began by selecting [Au_25_(*p*MBA)_18_]^−^ NCs as the model system for motif exchange and reconstruction with Cu-(*p*MBA) complexes, owing to their exceptional stability, atomically precise structure, and well-known structure-dependent electronic properties.^26–31^ The synthesis of [Au_25_(*p*MBA)_18_]^−^ NCs followed established protocols,^32,33^ in which carbon monoxide (CO) serves as the reductant for the Au­(I)-*p*MBA complex (see Methods for details). The as-synthesized protonated [Au_25_(*p*MBA)_18_]^−^ NCs dissolved in dimethylformamide (DMF) solution, forming a reddish-brown solution. The ultraviolet–visible (UV–vis) spectra exhibits the characteristic optical fingerprints of Au_25_(SR)_18_ NCs, including absorption bands at ca. 630 and 700 nm, attributed to the Au_13_ kernel, and at ca. 430 and 470 nm, associated with hybrid states involving the Au_2_(*p*MBA)_3_ surface motifs (Figure 1b). The molecular composition and charge state of [Au_25_(*p*MBA)_18_]^−^ NCs were verified by ESI-MS (Figure 1c), which reveals a series of distinct peaks corresponding to Au_25_(*p*MBA)_18_ NCs carrying 3–, 4–, and 5– charges within the *m*/*z* range of 1000–6000, respectively. The isotopic distribution of the [Au_25_(*p*MBA)_18_–2H]^3–^ species agrees closely with simulations, confirming accurate cluster assignment. By contrast, Cu-(*p*MBA) complexes form pale yellow DMF solutions and exhibit no distinctive UV–vis absorption features within the 400 to 1100 nm range (Figure S1), indicating their electronic neutrality and spectral transparency. These contrasting spectral behaviors provide a reliable basis for monitoring the transformation from [Au_25_(*p*MBA)_18_]^−^ to motif-edited AuCu nanoclusters.

We next performed surface motif editing by introducing Cu-(*p*MBA) complexes into [Au_25_(*p*MBA)_18_]^−^ nanocluster solution. Upon reaction, the solution color gradually shifted from reddish-brown to pale brown, signaling structural transformation. UV–vis spectroscopy revealed the disappearance of the ca. 430 and 470 nm absorption bands—previously assigned to hybrid kernel-motif transitions—while the kernel-associated bands at ca. 630 and 700 nm remained largely unaffected (Figure 1b). This selective spectral change indicates that the surface motifs were successfully reconstructed without disrupting the Au_13_ kernel, establishing a clear divergence between shell and kernel reconfiguration. The composition of the resulting AuCu nanoclusters was further confirmed by electrospray ionization mass spectrometry (ESI-MS). A dominant signal at *m*/*z* = 1550.9 (Figure 1d) corresponds to a 3– charged species, which matches well with the calculated isotopic pattern of [Au_13_Cu_4_(*p*MBA)_12_]^3–^, confirming its identity. Tandem mass spectrometry (MS/MS) analysis further verified the structural motifs. Upon increasing collision energy to 20 eV, a prominent fragment ion at *m*/*z* ≈ 2073 emerged, consistent with the loss of a single – Cu-(*p*MBA)_3_ unit (Figure 1e and Figure S2), thus identifying Cu-(*p*MBA)_3_ as a major surface motif in the reconstructed cluster. To explore the structural robustness and scope of motif exchange, we performed reactions under varied Cu feed ratios. At high [Cu]/[Au_25_] ratios (>18), complete transformation to [Au_3_Cu_2_(*p*MBA)_6_]^−^ was observed (Figures S3 and S4), highlighting the tunability of the exchange process and suggesting that [Au_13_Cu_4_(*p*MBA)_12_]^3–^ forms within a narrow compositional window. Together, the monoisotopic ESI-MS, isotopic simulations, and fragmentation analyses provide converging evidence that Cu-(*p*MBA)_3_ motifs are selectively integrated onto the Au_13_ kernel, replacing original Au_2_(*p*MBA)_3_ units without altering the kernel geometry.

To elucidate the structural configuration of [Au_13_Cu_4_(*p*MBA)_12_]^3–^ nanoclusters, we employed complementary spectroscopic and theoretical approaches. ^1^H nuclear magnetic resonance (^1^H NMR) spectroscopy (Figure 2a) revealed two distinct environments for the aromatic protons of chemically identical *p*MBA ligands, consistent with a symmetric arrangement of Cu-(*p*MBA)_3_ surface motifs on the Au_13_ kernel. A series of peaks corresponding to [Au_3_Cu_2_(*p*MBA)_6_]^−^ were also observed, suggesting the presence of a small amount of this species as a byproduct. This result is consistent with the ESI-MS data obtained from products synthesized at different [Cu]/[Au_25_] ratios. We then performed the thermogravimetric analysis (TGA) on freeze-dried purified cluster to further validate the composition and purity (Figure S5). The observed total mass loss of 34% in close agreement with the theoretical value of 40% for ligand dissociation in [Au_13_Cu_4_(*p*MBA)_12_]^3–^. This minor discrepancy (6%) confirms the presence of trace impurity, while robustly demonstrating that our target cluster is the predominant species. To address the purity of [Au_13_Cu_4_(*p*MBA)_12_]^3–^, we also compared the ^1^H NMR spectra and ESI-MS before and after purification (Figures S6 and S7). Despite these efforts, a trace amount of [Au_3_Cu_2_(*p*MBA)_6_]^−^ remains present. X-ray absorption fine structure (XAFS) analyses at both the Au L_3_-edge and Cu K-edge further resolved the coordination environments. The k^3^-weighted X-ray fine structure (EXAFS) spectra (Figure 2b,c) showed that Au atoms remained exclusively coordinated with sulfur, with no detectable Au–Cu scattering, thereby confirming the preservation of the Au_13_ kernel and the absence of alloy-type bonding. In [Au_13_Cu_4_(*p*MBA)_12_]^3–^, the Cu–S bond length (ca. 2.2 Å) was slightly shorter than that in free Cu-(*p*MBA) complexes (ca. 2.3 Å), suggesting stronger Cu–S interactions upon incorporation. Wavelet transform analysis and EXAFS fitting (Figure S8 and Table S1) further indicated an average Cu–S coordination number of ca. 3.3, pointing to under-coordinated, trigonal-planar Cu motifs. These spectroscopic results were corroborated by DFT simulations (Figure 2d–f, Figure S9, and Table S2), which confirmed a stable structure consisting of an icosahedral Au_13_ kernel capped by four Cu-(*p*MBA)_3_ units arranged tetrahedrally since it is difficult to crystalline water-soluble metal nanoclusters to obtain a crystal structure. Each Cu atom coordinates with three thiolates along the 3-fold symmetry axes of the icosahedron. The simulated bond lengths and coordination modes matched well with the experimental EXAFS data (Figures S10–S13) and were consistent with MS/MS evidence identifying Cu-(*p*MBA)_3_ as the dominant surface motif. Although single-crystal X-ray diffraction remains inaccessible for the hydrophilic thiolate-protected clusters, the convergence of spectroscopic, computational, and mass spectrometric evidence provides a strong support of the proposed [Au_13_Cu_4_(*p*MBA)_12_]^3–^ architecture.

**Figure 2:**
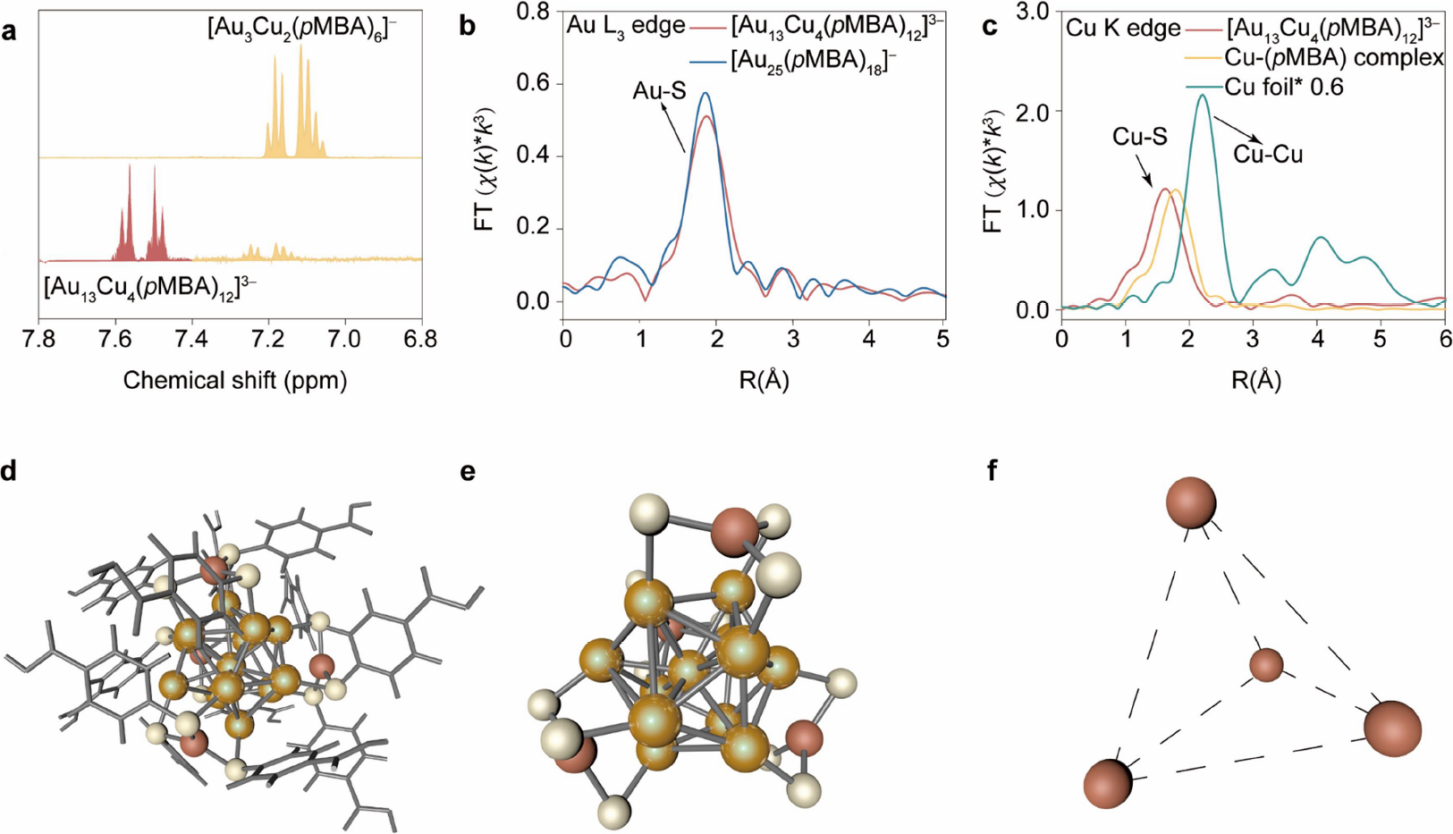
Structure correlation between [Au_25_ (*p*MBA)_18_]^−^ and [Au_13_Cu_4_(*p*MBA)_12_]^3–^ NCs. (a) ^1^H nuclear magnetic resonance spectra of [Au_13_Cu_4_(*p*MBA)_12_]^3–^ compared to [Au_3_Cu_2_(*p*MBA)_6_]^−^. (b) Fourier-transform k^3^-weighted Au L_3_ edge extended X-ray absorption fine structure (FT-EXAFS) spectra for [Au_25_(*p*MBA)_18_]^−^ and [Au_13_Cu_4_(*p*MBA)_12_]^3–^, showing the preservation of Au–S coordination upon motif editing. (c) FT-EXAFS analysis at Cu K-edge for Cu-(*p*MBA) complex and [Au_13_Cu_4_(*p*MBA)_12_]^3–^, confirming exclusive Cu–S coordination and the absence of Au–Cu bonding. (d–f), DFT-optimized structural models of [Au_13_Cu_4_(*p*MBA)_12_]^3–^, featuring an icosahedral Au_13_ kernel decorated with four Cu-(*p*MBA)_3_ motifs in trigonal planar configurations (yellow = Au, red = Cu, light yellow = S, gray stick = *p*MBA ligand).

To elucidate the transformation mechanism from [Au_25_(*p*MBA)_18_]^−^ into [Au_13_Cu_4_(*p*MBA)_12_]^3–^, we propose a three-step surface motif editing process as follows (Schematic illustration in Figure S14): (i) coordination of Cu-(*p*MBA) complexes onto the Au NC surface; (ii) cleavage of native Au_2_(*p*MBA)_3_ motifs; and (iii) substitution by Cu-(*p*MBA)_3_ motifs. This kernel-preserving transition was monitored in real time using *in situ* UV–vis spectroscopy and ESI-MS. The UV–vis absorption peaks at ca. 430 and 470 nm, associated with kernel-motif interactions, disappear within 5 min, while signals at ca. 630 and 700 nm from the Au_13_ kernel remain largely unchanged, indicating rapid motif reconstruction without disruption of the metal kernel (Figure 3a). Simultaneously, real-time ESI-MS (Figure 3b and Figure S15) reveals the immediate emergence of [Au_13_Cu_4_(*p*MBA)_12_]^3–^ accompanied by transient intermediates such as [Au_13_Cu_7_(*p*MBA)_9_–2H]^3–^ (*m*/*z* = 1460), [Au_14_Cu_6_(*p*MBA)_9_]^3–^ (*m*/*z* = 1506.1), and [Au_3_Cu_2_(*p*MBA)_6_]^−^ (*m*/*z* = 1636.7) (Figures S16–S18). These species dominate after 1 h but eventually converge toward a compositionally focused final product: [Au_13_Cu_4_(*p*MBA)_12_]^3–^, with minor residual amounts of [Au_3_Cu_2_(*p*MBA)_6_]^−^ persisting beyond 4 h. This temporal evolution illustrates a stepwise, ligand-mediated transformation pathway governed by surface coordination dynamics and composition refinement.

**Figure 3:**
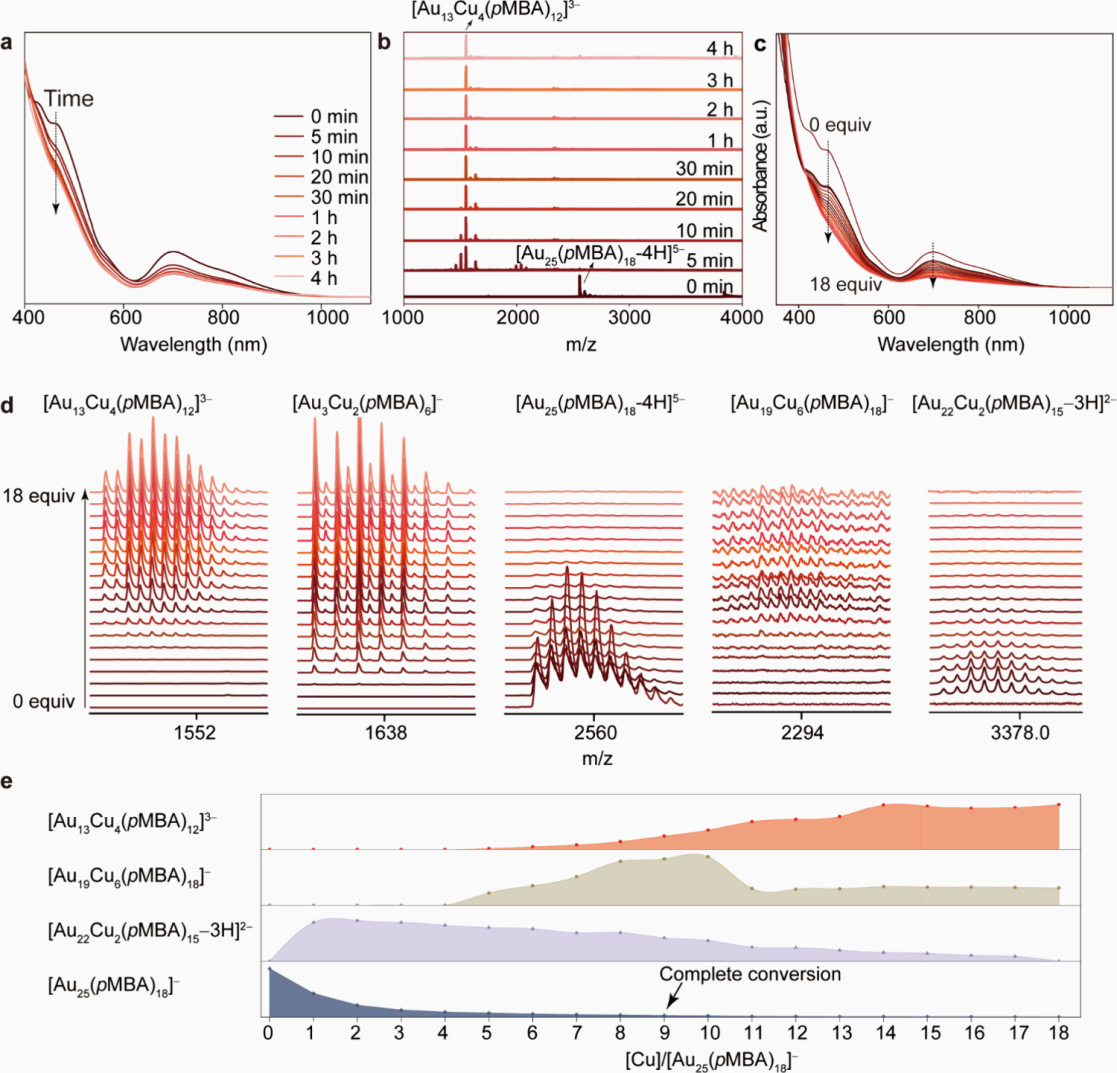
Elucidating the transformation pathway from [Au_25_(*p*MBA)_18_]^−^ to [Au_13_Cu_4_(*p*MBA)_12_]^3–^ via stepwise surface motif editing. (a) Time-resolved UV–vis absorption spectra showing the disappearance of kernel-motif hybrid bands at ca. 430 and 470 nm and preservation of Au_13_ kernel signals at ca. 630 and 700 nm, indicating motif transformation without kernel disruption. (b) Corresponding *in situ* ESI-MS tracking reveals the rapid formation of [Au_13_Cu_4_(*p*MBA)_12_]^3–^ within 5 min, accompanied by transient intermediates. (c) UV–vis spectra of the stepwise reaction reveals a progressive decline in motif-associated absorptions with incremental Cu-(*p*MBA) addition. (d) ESI-MS analysis uncovers the formation of distinct intermediates, and (e) Corresponding peak intensity profiles of precursors and intermediates.

To gain further mechanistic insight into the motif editing process, we carried out a stepwise titration of Cu-(*p*MBA) complexes into [Au_25_(*p*MBA)_18_]^−^ NC solutions and monitored the evolution using UV–vis spectra and ESI-MS. The characteristic absorption bands at 462, 630, and 700 nm progressively diminished upon incremental Cu addition (Figure 3c). After 9 equiv, the 462 nm motif-associated band nearly vanished, while the Au_13_-kernel signals at ca. 630 and 700 nm were retained. Beyond 18 equiv, however, all optical features disappeared, indicating potential etching or degradation, likely due to excessive Cu-induced motif disruption. Parallel ESI-MS tracking (Figure 3d,e and Figure S19) revealed a series of well-resolved intermediate species whose intensity evolved in a dose-dependent manner. Specifically, we observed transient accumulation of [Au_28_Cu_2_(*p*MBA)_24_]^3–^, [Au_22_Cu_2_(*p*MBA)_15_–3H]^2–^, and [Au_19_Cu_6_(*p*MBA)_18_]^−^, each rising and then diminishing as Cu equivalents increased. (Figures S20–S22) These trends confirm their role as kinetic intermediates on route to the final [Au_13_Cu_4_(*p*MBA)_12_]^3–^ product. The early emergence of [Au_28_Cu_2_(*p*MBA)_24_]^3–^, a likely adduct of [Au_25_(*p*MBA)_18_]^−^ and [Au_3_Cu_2_(*p*MBA)_6_]^−^. underscores a cluster–cluster association mechanism mediated by Cu-ligand interactions (Figure S23). Notably, [Au_3_Cu_2_(*p*MBA)_6_]^−^ and its dimer [Au_6_Cu_4_(*p*MBA)_12_]^−^ persisted as minor side products, reinforcing their structural compatibility with the final AuCu NC (Figures S24 and S25). Together, these spectroscopic and mass spectrometric observations delineate a well-defined transformation landscape, wherein Cu-mediated motif reconstruction proceeds through sequential substitution and composition focusing, while maintaining the integrity of the Au_13_ kernel.

To establish the necessity of the surface motif editing strategy, we compared it against conventional coreduction synthesis of AuCu alloy nanoclusters.^34–36^ Unlike our motif-directed approach, coreduction of HAuCl_4_ and CuCl_2_ in the presence of *p*MBA ligands yielded clusters that retained the signature UV–vis features of [Au_25_(*p*MBA)_18_]^−^, with only a slight red shift from 700 to 707 nm (Figure S26). This suggests that Cu dopants were incorporated into the kernel without affecting the Au_2_(*p*MBA)_3_ surface motifs, a result corroborated by ESI-MS analysis showing [Au_25–*x*_Cu_*x*_(*p*MBA)_18_]^−^ NCs (*x* = 0, 1) compositions (Figure S27). However, increasing the Cu/Au ratio beyond 10% led to the collapse of the M_25_(SR)_18_ (M = Au or Cu) framework, evidenced by loss of characteristic absorptions (Figure S28), further confirming the structural instability of uncontrolled alloying. Crucially, replacing Cu-(*p*MBA) complex with bare Cu^2+^ ions failed to trigger motif exchange (Figure S29), underscoring the role of Cu-SR complexation in facilitating motif-level selectivity through sulfur–gold affinity. To probe the generality of our strategy, we extended the motif editing protocol to a larger thiolate-protected gold cluster, [Au_38_(*p*MBA)_24_]^0^, which features a bi-icosahedral Au_23_ kernel capped with six Au_2_(SR)_3_ staples. Upon treatment with Cu-(*p*MBA) complexes, UV–vis and ESI-MS analyses confirmed the successful formation of [Au_26_Cu_7_(*p*MBA)_22_]^3–^, consistent with a complete substitution of the six dimeric Au_2_(SR)_3_ motifs by Cu-(SR)_3_ units (Figures S30–S33). This extension illustrates the robustness and modularity of our motif-by-motif editing approach across structurally distinct nanoclusters

To assess how surface motif editing modulates the exposure and reactivity of kernel active sites, we employed the hydrogen evolution reaction (HER) as a model system. Electrocatalytic performances of [Au_25_(*p*MBA)_18_]^−^ and the edited [Au_13_Cu_4_(*p*MBA)_12_]^3–^ clusters were evaluated using rotating disk electrode (RDE) measurements in 0.5 M H_2_SO_4_. All potentials were referenced to the reversible hydrogen electrode (RHE). [Au_13_Cu_4_(*p*MBA)_12_]^3–^ exhibits dramatically enhanced HER performance, requiring overpotentials of only 206 and 345 mV to reach current densities of 10 and 50 mA cm^–2^, respectively, much lower than those of [Au_25_(*p*MBA)_18_]^−^ (640 and 855 mV) (Figures S34 and S35). Importantly, the performance of [Au_13_Cu_4_(*p*MBA)_12_]^3–^ surpasses that of bulk Au electrodes and residual species such as [Au_3_Cu_2_(*p*MBA)_6_]^−^, confirming that the improvement arises from structural optimization rather than Cu content alone. We also compared the LSV of [Au_13_Cu_4_(*p*MBA)_12_]^3–^ before and after purification to eliminate the contribution of [Au_3_Cu_2_(*p*MBA)_6_]^−^ to HER performance (Figure S36). Indeed, Cu-(*p*MBA) complexes with higher Cu loading (25% more Cu) exhibited negligible HER activity, ruling out a direct catalytic contribution from Cu sites. Electrochemical impedance spectroscopy (EIS) further confirmed the superior charge transfer characteristics of [Au_13_Cu_4_(*p*MBA)_12_]^3–^ over the parent cluster (Figure S37). In parallel, coreduction synthesized [Au_25‑*x*_Cu_*x*_(*p*MBA)_18_]^−^ NCs (*x* = 0, 1) clusters showed substantially inferior activity (Figure S38), reinforcing the unique role of surface motif editing. To probe the physical origin of this enhancement, we quantified the electrochemically active surface area (ECSA). The [Au_13_Cu_4_(*p*MBA)_12_]^3–^ clusters exhibited a 16-fold higher double-layer capacitance (1.88 vs 0.124 mF cm^–2^), indicative of greater kernel accessibility (Figures S39–S41). This translated to over 300-fold enhancement in Au-mass-normalized activity and a 180-fold increase in turnover frequency (TOF) at 10 mA cm^–2^ (Figures S42 and S43).

To gain mechanistic insights into how motif editing enhances HER kinetics, we analyzed the rate-determining step (RDS) using Tafel slope measurements. Compared to [Au_25_(*p*MBA)_18_]^−^, [Au_13_Cu_4_(*p*MBA)_12_]^3–^ cluster exhibits a lower Tafel slope (82 vs 139 mV dec^–1^), indicating a transition of the RDS from a Volmer step to a mixed Volmer-Heyrovsky regime (Figure 4a). To further decipher the origin of this enhancement, *in situ* shell-isolated nanoparticle-enhanced Raman spectroscopy (SHINER) was employed to monitor intermediate species during HER. Under – 0.3 V, [Au_13_Cu_4_(*p*MBA)_12_]^3–^ exhibits a distinct Raman band at ca. 1659 cm^–1^, attributable to Au–H stretching, which intensifies with increasing overpotential.^37^ In contrast, no such Au–H signal is observed for the parent [Au_25_(*p*MBA)_18_]^−^ cluster (Figure 4b and Figures S44–S46), suggesting that surface reconstruction increases the accessibility and reactivity of Au kernel sites. DFT calculations support this conclusion. Upon motif editing, the H adsorption free energy on the Au_3_ facet drops from 0.95 to 0.03 eV, indicating more favorable hydrogen binding (Figure 4c,d). In comparison, Cu sites present less favorable adsorption with ΔG = 0.15 eV, confirming that Au remains the principal active site. After motif editing, the yielded [Au_13_Cu_4_(*p*MBA)_12_]^3–^ NCs demonstrate higher a prior comprehensive HER performance that outperform [Au_25_(*p*MBA)_18_]^−^ (Figure 4e). Together, Tafel analysis, *in situ* Raman, and DFT form a consistent picture: the Cu-assisted motif reconstruction opens previously inaccessible Au_3_ facets, thereby accelerating HER kinetics through improved proton adsorption and a shift in rate-limiting steps. Our findings establish surface motif editing as a mechanistically distinct strategy from composition doping, achieving catalytic enhancement not by introducing new active centers, but by geometrically liberating the existing ones. This challenges the conventional trade-off between structural stability and catalytic accessibility in nanoclusters, opening a path toward kernel-protected yet catalytically open architectures.

**Figure 4:**
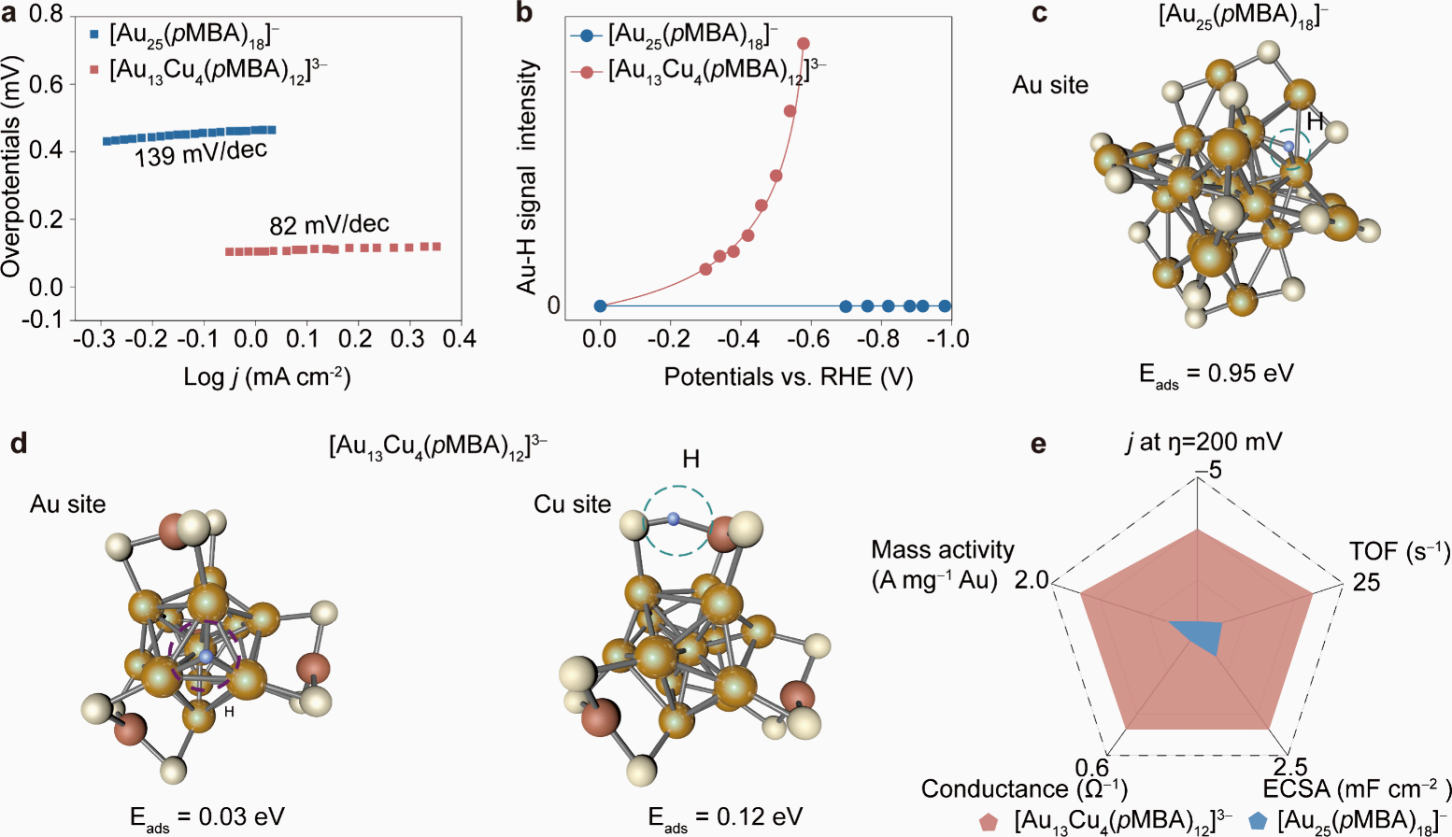
Electrocatalytic HER performance of [Au_25_(*p*MBA)_18_]^−^ and [Au_13_Cu_4_(*p*MBA)_12_]^3–^ NCs. (a) Tafel plots derived from LSV curves, highlighting enhanced HER kinetics upon motif editing. (b) *In situ* shell-isolated nanoparticle enhanced Raman spectroscopy (SHINER) showing the potential-dependent intensity of the Au–H stretching vibration in [Au_13_Cu_4_(*p*MBA)_12_]^3–^. DFT-optimized structure with calculated H* adsorption energy of (c) [Au_25_(*p*MBA)_18_]^−^ (active sites: Au_3_ facets) and (d) of [Au_13_Cu_4_(*p*MBA)_12_]^3–^ (active sites: Au and Cu center) (Ligands omitted for clarity, color codes: yellow = Au, red = Cu, light yellow = S, blue = H). (e) Comparative performance metrics including current density at η = 200 mV, Au-normalized mass activity, turnover frequency, and electrical conductance, underscoring the synergistic enhancement in catalytic activity upon motif editing.

To assess the structural robustness of the [Au_13_Cu_4_(*p*MBA)_12_]^3–^ nanocluster under electrochemical operation, we performed long-term chronopotentiometry (CP) and X-ray absorption spectroscopy (XAS). The cluster maintained a stable current density of 60 mA cm^–2^ over 60 h of hydrogen evolution reaction (HER, Figure 5a), indicating robust electrochemical durability. To probe structural integrity at the atomic level, we conducted X-ray absorption near-edge structure (XANES) and EXAFS analyses at both Au L_3_- and Cu K-edges before and after HER (Figure 5b–e). The spectra revealed no discernible changes in local coordination environments or oxidation states, confirming that both Au and Cu centers remain structurally intact throughout catalytic operation. We attribute this exceptional stability to two key structural features: (i) the closed-shell electronic configuration of the icosahedral Au_13_ kernel ((1S)^2^(1P)^6^), which imparts inherent thermodynamic stability; (ii) the trigonal-planar Cu–S surface motifs, in which each Cu^+^ center is coordinated to three thiolates (CN = 3) from *p*MBA with nearly identical bond lengths (2.24–2.27 Å), yielding a spatially compact and electronically robust surface geometry (Figure 5f). This geometry stands in stark contrast to the linear Au_2_(*p*MBA)_3_ motifs (CN = 2) of the parent cluster, and confers multiple advantages: higher coordination number, improved Cu–S covalency, and reduced steric coverage on the Au_13_ kernel. The minimal bond length variation (<0.03 Å) further reflects the rigidity of the Cu–S framework, which effectively insulates the kernel from distortion or reductive degradation. In addition, we compared the UV–vis spectra of the cluster before and after the hydrogen evolution reaction (HER), as shown in Figure S47a. The characteristic absorption peak at approximately 699 nm, assigned to the Au_13_ kernel, remains virtually unchanged after HER. This strongly indicates that both the electronic structure and the kernel configuration of the cluster are preserved during the electrocatalytic process. To further confirm the structural integrity of the cluster, we conducted ESI-MS measurements on the post-HER redissolved samples (Figure S47). The results clearly show no evidence of ligand dissociation or kernel fragmentation, as demonstrated by the identification of the intact species [Au_13_Cu_4_(*p*MBA)_12_]^3–^. Overall, the motif-edited cluster [Au_13_Cu_4_(*p*MBA)_12_]^3–^ not only exhibits enhanced catalytic performance but also maintains high structural and compositional stability.

**Figure 5:**
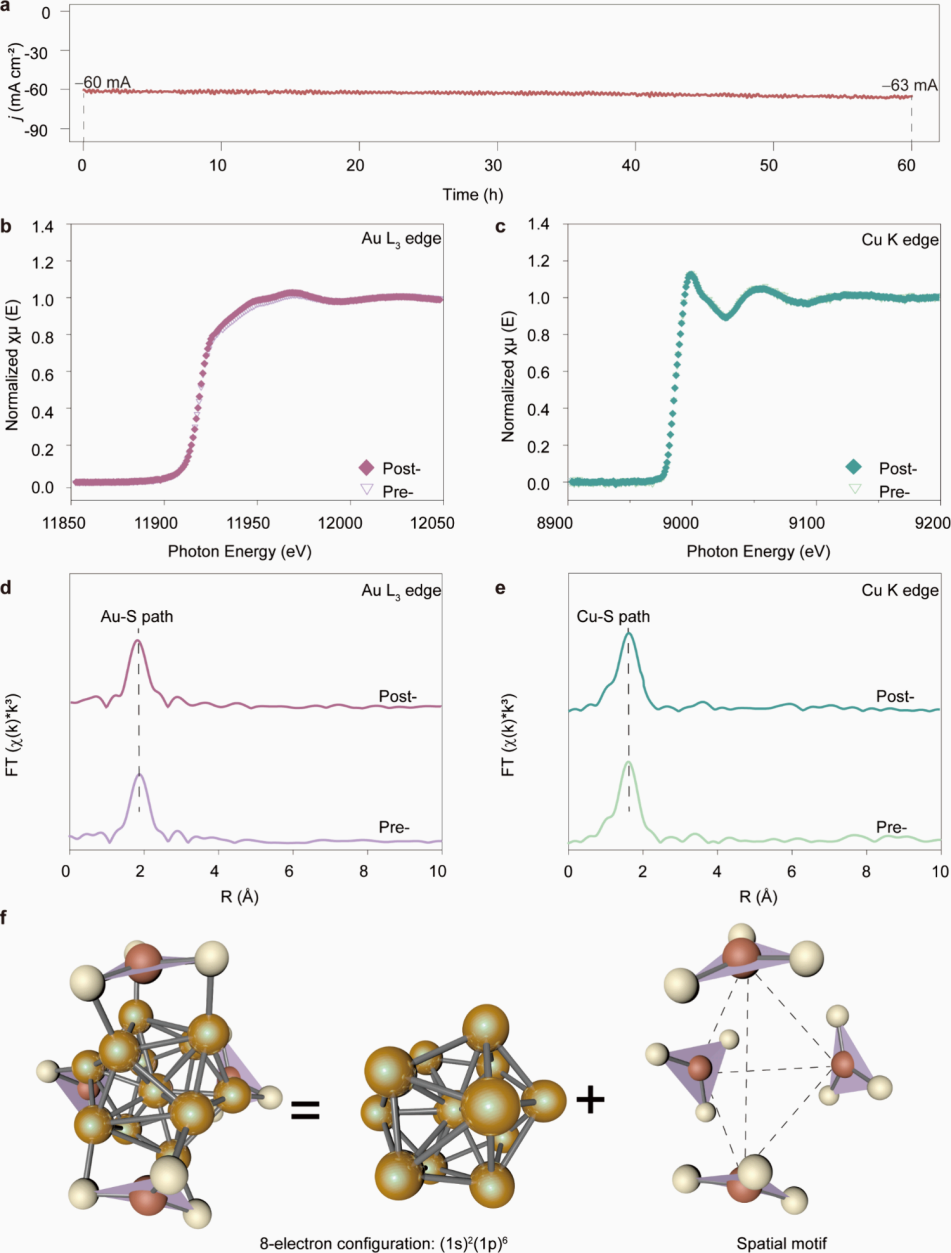
Structural and electrochemical stability of [Au_13_Cu_4_(*p*MBA)_12_]^3–^ NCs during HER. (a) Chronoamperometric stability test at – 0.32 V (vs RHE) over 60 h, showing consistent current density. (b, c), Au L_3_-edge and Cu K-edge XANES spectra of [Au_13_Cu_4_(*p*MBA)_12_]^3–^ recorded pre- and post-HER, indicating unchanged oxidation states. (d, e) Corresponding FT-EXAFS spectra at the Au and Cu edges, confirming retention of local bonding environments. (f) DFT optimized structure of [Au_13_Cu_4_(*p*MBA)_12_]^3–^, showing trigonal-planar Cu-(*p*MBA)_3_ surface motifs anchored on the icosahedral Au_13_ kernel (ligands omitted for clarity; color code: green = Au, blue = Cu, yellow = S; triangles mark Cu-(*p*MBA)_3_ motifs).

## Conclusions

In summary, we presented a motif-by-motif surface editing strategy that enables atomically precise exposure of catalytically active metal kernels while preserving the structural integrity of the kernel. By rationally replacing sterically hindered Au_2_(*p*MBA)_3_ motifs with compact Cu-(*p*MBA)_3_ units on [Au_25_(*p*MBA)_18_]^−^ nanoclusters, we achieved [Au_13_Cu_4_(*p*MBA)_12_]^3–^ species with a more open and symmetric surface, exposing 16 accessible Au_3_ facets compared to only 2 in the parent cluster. We show that such architectural reconfiguration enhances hydrogen evolution performance, achieving a 10 mA cm^–2^ overpotential of 206 mV and a turnover frequency of 18.8 s^–1^, representing a 434-mV suppression and 180-fold improvement, respectively. Mechanistic insights from *in situ* mass spectrometry and X-ray absorption spectroscopy confirm a stepwise motif exchange pathway and a preserved Au_13_ kernel during reconstruction. Our approach presents a conceptually distinct route from conventional ligand or atom exchange, offering a structurally coherent means to engineer site accessibility without compromising atomic precision. Looking forward, this strategy may open avenues for surface-programmed catalysis in atomically defined nanoclusters, with potential generalization to multimetallic motifs and broader electrochemical transformations.

## Supplementary Material



## Notes

Synthesis and purification: gold nanoclusters, copper-pMBA complex, and gold–copper nanoclusters; electrochemical characterization: linear sweep voltammetry (LSV), cyclic voltammetry (CV), electrochemical impedance spectroscopy (EIS), determination of electrochemical active surface area (ECSA) via double-layer capacitance, and assessment of specific activity and turnover frequency (TOF); spectroscopic analysis: electrospray ionization mass spectrometry (ESI-MS) with intensity evolution analysis, UV–vis absorption spectroscopy, and *in situ* Raman spectroscopy; material characterization: thermogravimetric analysis (TGA), ^1^H NMR spectrometry; computational details: density functional theory (DFT) calculations; and structural analysis: wavelet transform analysis of Cu K-edge and Au L_3_ edge EXAFS, EXAFS fitting, and DFT-optimized structural modeling (PDF)
