# Supplementary material for: Motif Editing Reveals Hidden Active Sites in Atomically Precise Metal Nanoclusters for Enhanced Electrocatalysis
Source: J Am Chem Soc. 2025 Oct 23;147(44):40205–13. doi: 10.1021/jacs.5c08684 (PMC12593332; doi:10.1021/jacs.5c08684)
Supplement: Supplementary file 1 [file ja5c08684_si_001.pdf]

SUPPLEMENTARY INFORMATION

Motif Editing Reveals Hidden Active Sites in Atomically Precise Metal Nanoclusters for Enhanced Electrocatalysis

Zhihe Liu[a,g,i], Moshuqi Zhu[b,h,i], Bo Li[d,i], Junmei Chen[c], Shibo Xi[e,*], Yang-Yang Yu[f,*], Lu Xia[c], Lei Wang[a,g,*], De-en Jiang[d,*], F. Pelayo García de Arquer[c,*], and Jianping Xie[a,g,h*]

[a] Department of Chemical and Biomolecular Engineering, National University of Singapore, 117585, Singapore

[b] School of Chemical Engineering and Technology, Tianjin University, Tianjin 300072, China

[c] ICFO – Institut de Ciències Fotòniques, The Barcelona Institute of Science and Technology, 08860 Castelldefels, Barcelona, Spain

[d] Department of Chemical and Biomolecular Engineering, Vanderbilt University, Nashville, Tennessee, 37212, United States

[e] Institute of Sustainability for Chemicals, Energy and Environment (ISCE2), Agency for Science, Technology and Research (ASTAR), 627833, Singapore

[f] Information Materials and Intelligent Sensing Laboratory of Anhui Province, Institutes of Physical Science and Information Technology, Anhui University, Hefei 230601, China

[g] Centre for Hydrogen Innovations, National University of Singapore, 1 Engineering Drive 3, Singapore 117585, Singapore

[h] Joint School of National University of Singapore and Tianjin University International Campus of Tianjin University Binhai New City, Fuzhou 350207, P. R. China

[i] These authors contributed equally: Zhihe Liu; Moshuqi Zhu; Bo Li

Experimental details

Chemicals and materials: Gold tetrachloride trihydrate ($HAuCl_4 \cdot 3H_2O$), copper(II) chloride dihydrate ($CuCl_2 \cdot 2H_2O$) *para*-mercaptobenzoic acid (*p*MBA), sodium hydroxide (NaOH), N-methyl-2-pyrrolidone (NMP), tetrahydrofuran (THF), and N, N-dimethylformamide (DMF) were purchased from Sigma-Aldrich. Ethanol was purchased from Fisher. Acetic acid (HOAc) was purchased from Merck. All chemicals were commercially available without further purification. Carbon monoxide (CO) with the purity of 99% and hydrogen with the purity of 99.99% without further purification was provided by *Singapore Oxygen Air Liquide Pte Ltd* (SOXAL). Carbon powder (Vulcan XC-72) was purchased from Fuel Cell Store. Ultrapure Millipore water (18.2 MΩ) was used for preparation on all aqueous solutions. All glassware was washed with aqua regia, then rinsed with ultrapure water before use.

Methods

Synthesis of $[Au_{25}(pMBA)_{18}]^-$

$[Au_{25}(pMBA)_{18}]^-$ NCs were prepared by using CO as a reducing agent according to our previously reported protocol.[1] In particular,10 mL of aqueous solution of 50 mM *p*MBA (in 150 mM NaOH) and 5 mL of 50 mM $HAuCl_4$ were added into 238.75 mL of ultrapure water sequentially. Then, the mixed solution was stirred for 5 min. After that, the pH of the reaction mixture was brought up to 11.05 by dropping in 1.0 M NaOH aqueous solution and then was stirred for another 10 min to form Au(I)-*p*MBA complexes. subsequentially, CO was bubbled into the reaction mixture for 2 min to initiate the reduction of Au(I)-*p*MBA complexes. The mixed solution was allowed for proceeding air-tightly for 7 days at room temperature (25 °C) and under vigorous stirring. The reddish-brown solution was obtained at the end of this procedure as raw products. The raw product solution was concentrated by rotary evaporation (water bath at 45 °C, cooling at 2 °C, rotation rate of 100 rpm). The concentrated solution was then mixed with twice its volume of ethanol and centrifuged at 11,500 rpm for 5 min. After discarding the supernatant, the precipitate was collected and washed with DMF and

ethanol. To dissolve $[Au_{25}(pMBA)_{18}]^-$ NCs in DMF, $[Au_{25}(pMBA)_{18}]^-$ NCs were protonated with acetic acid (HOAc). Specifically, the $[Au_{25}(pMBA)_{18}]^-$ precipitate was first recovered and treated with 4 mL of HOAc in 30 vol.% DMF. Subsequently, the protonated $[Au_{25}(pMBA)_{18}]^-$ NC were precipitated by centrifugation at 11,000 rpm for 5 minutes with 16 mL toluene. The obtained precipitates were then redissolved in 4 mL of HOAc in DMF (10 vol. %), followed by a centrifugation for 5 min with 16 mL toluene. After that, the purified $[Au_{25}(pMBA)_{18}]^-$ NC precipitate was redissolved in DMF for further surface exchange reaction.

Preparation of Cu-(*p*MBA) complex

Cu(I)-(*p*MBA) complexes with a feeding *p*MBA-to-Cu(II) ratios ($R_{pMBA/Cu}$) of 3:1 were prepared by mixing 2.4 mL of aqueous solution of *p*MBA ligands (in 0.1 M NaOH) with an aqueous solution of $CuCl_2$ (2 mL, 20 mM), followed by the addition of 44 μL of HOAc. The resulting protonated Cu(I)-(*p*MBA) complex was then isolated by centrifuging at 11,000 rpm for 5 min, after which the precipitate was redissolved in 2.4 mL of DMF.

Synthesis of $[Au_{13}Cu_4(pMBA)_{12}]^{3-}$ NCs

$[Au_{13}Cu_4(pMBA)_{12}]^{3-}$ NCs were synthesized by mixing DMF solutions of protonated $[Au_{25}(pMBA)_{18}]^-$ NCs and Cu-(*p*MBA) complexes. Typically, 2 mL of 0.04 mM $[Au_{25}(pMBA)_{18}]^-$ NCs were added into 43.17 μL of Cu(I)-(*p*MBA) complexes. The reaction mixture was then stirred continuously for 4 h, yielding a pale-brown solution of $[Au_{13}Cu_4(pMBA)_{12}]^{3-}$ NCs. The synthesized $[Au_{13}Cu_4(pMBA)_{12}]^{3-}$ NCs were purified by precipitation via centrifugation at 11,000 rpm for 5 minutes using twice its volume of toluene. The supernatant, which contained the major impurities, was carefully removed. The resulting precipitate, primarily consisting of the target $[Au_{13}Cu_4(pMBA)_{12}]^{3-}$ NCs was then redissolved in DMF for subsequent ink preparation.

Synthesis of $[Au_3Cu_2(pMBA)_6]^-$ NCs

The synthesis of $[Au_3Cu_2(pMBA)_6]^-$ NCs is similar to that of

$[Au_{13}Cu_4(p\text{MBA})_{12}]^{3-}$ NCs, with the primary difference being the use of 345.13 μL rather than of 43.17 μL of Cu(I)-(*p*MBA) complexes.

Synthesis of $[Au_{38}$ $(p\text{MBA})_{24}]^0$ NCs

The synthetic protocol of $[Au_{38}(p\text{MBA})_{24}]^0$ follows our previously reported seed-mediated synthesis approach.[2] In particular, First, 0.5 mL of a 50 mM ethanolic solution of *p*MBA was mixed with 0.25 mL of a 50 mM aqueous solution of $HAuCl_4$ in 9 mL of ultrapure water. The mixture was stirred for 5 min. Next, 1 M NaOH was added dropwise to the suspended Au(I)-(*p*MBA) complexes to adjust the pH to 12.4, followed by an additional 30 min of stirring. Afterward, 0.25 mL of $[Au_{25}(p\text{MBA})_{18}]^-$ aqueous solution was added, followed by bubbling in CO for 2 min. The reaction is stirred at room temperature for 6 days, yielding a black-brown solution. The purification and transfer to DMF are carried out similarly to the $[Au_{25}(p\text{MBA})_{18}]^-$ protocol.

Electrochemical experimental procedures

All electrochemical measurements were conducted at room temperature using a standard three-electrode system on an Auto lab electrochemical workstation. An Ag/AgCl electrode served as the reference, and a graphite rod was used as the counter electrode. A rotating disk electrode (RDE) with a glassy carbon surface was employed as the working electrode, with a rotation speed set to 1600 rpm. The electrolyte (0.5 M H_2SO_4) was purged with pure H_2 for at least 30 min to ensure H_2 saturation before testing. All potentials were IR-compensated and converted to the reversible hydrogen electrode (RHE) scale. Cyclic voltammetry (CV) and linear sweep voltammetry (LSV) were performed at scan rates of 50 mV s^{-1} and 5 mV s^{-1}, respectively. Electrochemical impedance spectroscopy (EIS) measurements were conducted over a frequency range of 100 kHz to 1 Hz at different potentials. The catalyst ink was prepared as follows: for NCs, the ink was prepared by mixing 0.5 mL NMP containing 2 mg carbon black with 0.5 mL of NCs (0.5 μmol/mL based on NC concentration) DMF solution and 20 μL of 5 wt% Nafion solution. In a typical process of preparing the working electrode, 10 μL of the catalyst ink was loaded onto a glassy carbon electrode (0.07 cm^2).

Computational Details

Spin-polarized density functional theory (DFT) calculations were performed in the Quickstep module[3] of CP2K.[4] The mixed Gaussian and plane wave (GPW) approach[5] using the Perdew-Burke-Ernzerhof (PBE) functional[6] was employed with the Grimme D3-dispersion correction.[7] The Kohn-Sham orbitals were expanded in the molecularly optimized basis set with a double-ζ Gaussian basis set augmented with a set of p-type polarization functions (MOLOPT-SR-DZVP)[8] with core electrons represented by the Goedecker-Teter-Hutter pseudopotentials.[9-11] In all calculations, the plane-wave kinetic energy cutoff was set to 400 Ry. Convergence threshold of the electronic structure relaxation was set to 10^{-6} Hartree and the force convergence criterion of the geometry optimizations was set to 4.5×10^{-4} Hartree/Bohr. The initial structure of $[Au_{13}Cu_4(p\mathrm{MBA})_{12}]^{3-}$ was constructed by placing one Cu-$(p\mathrm{MBA})_3$ motifs on each top of the four Au_3 facets of the Au_{13} kernel with Au-S distance set to 2.5 Å. The orientation of *p*MBA ligands were adjusted to ensure they were separated by at least 4 Å in the initial structure.

The computational hydrogen electrode[12] was used to obtain the Gibbs free energy of HER in the form of $\Delta G_{H*} = \Delta E_{H*} + \Delta E_{ZPE} - T\Delta S_H$, in which ΔE_{H*} is the hydrogen adsorption energy, ΔE_{ZPE} the difference of zero-point energy between adsorbed H* and the gas phase H_2. ΔS_H is the change of entropy from the gas phase H to the adsorbed H*, which is approximately half the entropy of gas-phase H_2 under standard condition.

Materials characterization

The pH of solutions and electrolytes was monitored using a Mettler Toledo FE 20 pH meter. The crude product was centrifuged with an Eppendorf Centrifuge 5424. UV-vis absorption spectra were acquired on a Shimadzu UV-1800 spectrometer in fast scan and single scan mode. X-ray photoelectron spectroscopy (XPS) was performed using a Kratos AXIS Ultra DLD spectrometer, with all binding energies referenced to the C(1s) hydrocarbon peak at 284.5 eV. Cluster concentrations were measured and normalized using inductively coupled plasma optical emission spectrometry (ICP-OES) on a Thermo Scientific iCAP 6000. Electrospray ionization mass spectrometry (ESI-MS)

was conducted in negative ion mode on a Bruker micro TOF-Q system. Detailed ESI-MS conditions were as follows: source temperature, 120 °C; dry gas flow rate, 4 L/min; nebulizer pressure, 0.4 bar; and capillary voltage, 3.5 kV. An *in-situ* Raman study was performed using a confocal microscopic Raman spectrometer (LabRam HR, Horiba, France). To enhance signal intensity, we employed shell-isolated nanoparticle-enhanced Raman spectroscopy (SHINERS), a strategy developed by Tian's group.[13] Specifically, 55 nm Au nanoparticles coated with a 2 nm SiO_2 shell (SHINs) were synthesized following their protocol. The surface-enhanced Raman spectroscopy (SERS) effect and the integrity of the SiO_2 shell were confirmed using pyridine as a probe molecule. The Raman tests were conducted with a 785 nm laser for excitation, and the ND filter was set to 10%, with an acquisition time of 10 seconds. X-ray absorption fine structure (XAFS) spectroscopy was conducted at the XAFCA beamline of the Singapore Synchrotron Light Source in fluorescence mode. Cu K-edge and Au L_3-edge XAFS spectra were collected using an electron energy of 0.7 GeV. The X-ray absorption near-edge structure (XANES) and extended X-ray absorption fine structure (EXAFS) spectra were analyzed with the Athena and Artemis software included in the Demeter package.[14] Cu foil and Au foil reference standards were measured in parallel for energy calibration.

Supplemental Figures

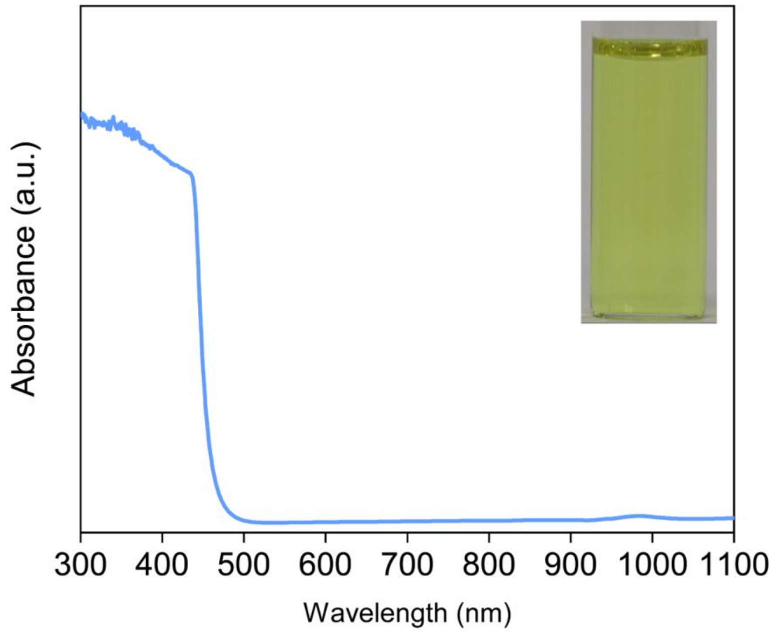

Figure S1. UV-vis absorption spectra of Cu-(*p*MBA) complexes.

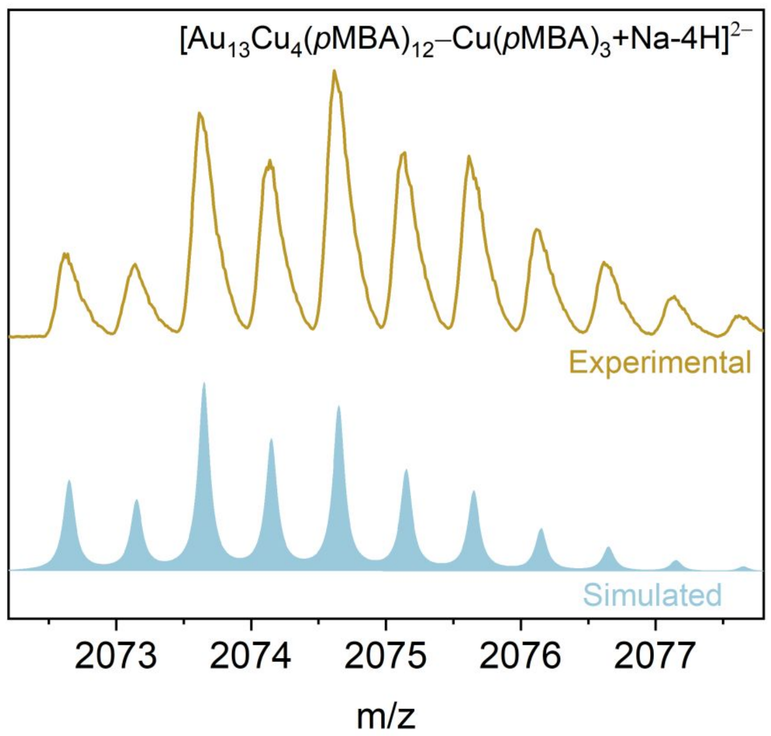

Figure S2. ESI-MS spectra of the emerging new species in tandem MS analysis of $[Au_{13}Cu_4(p\text{MBA})_{12}]^{3-}$ NCs at the isotope resolution. The filled patterns indicate the simulated isotope distribution of the labeled cluster species.

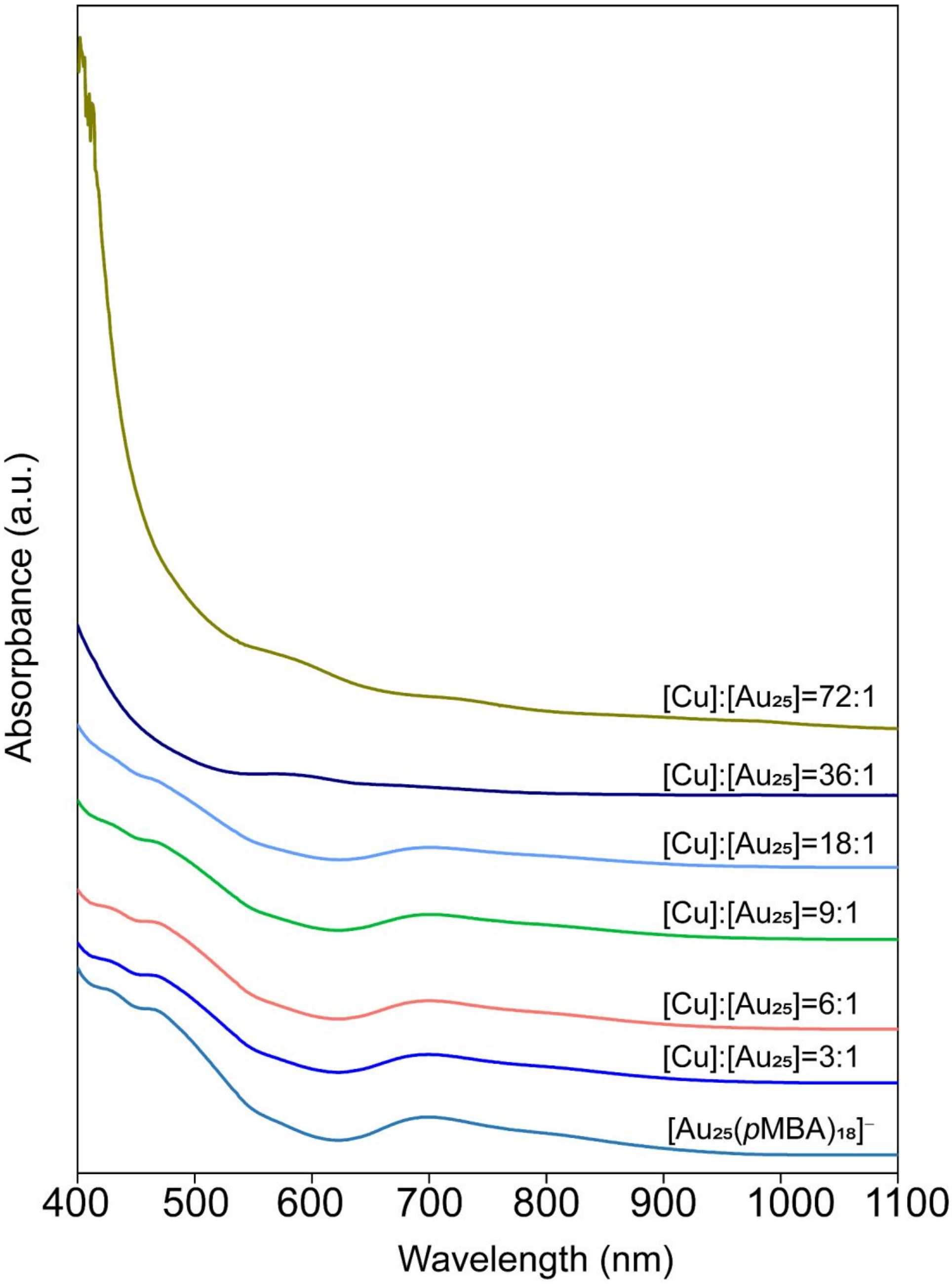

Figure S3. UV-vis absorption spectra of mixed solution of $[Au_{25}(pMBA)_{18}]^-$ NCs with Cu-(*p*MBA) complexes at different ratios of [Cu]/ $[Au_{25}]$. The reaction was allowed for 4 h.

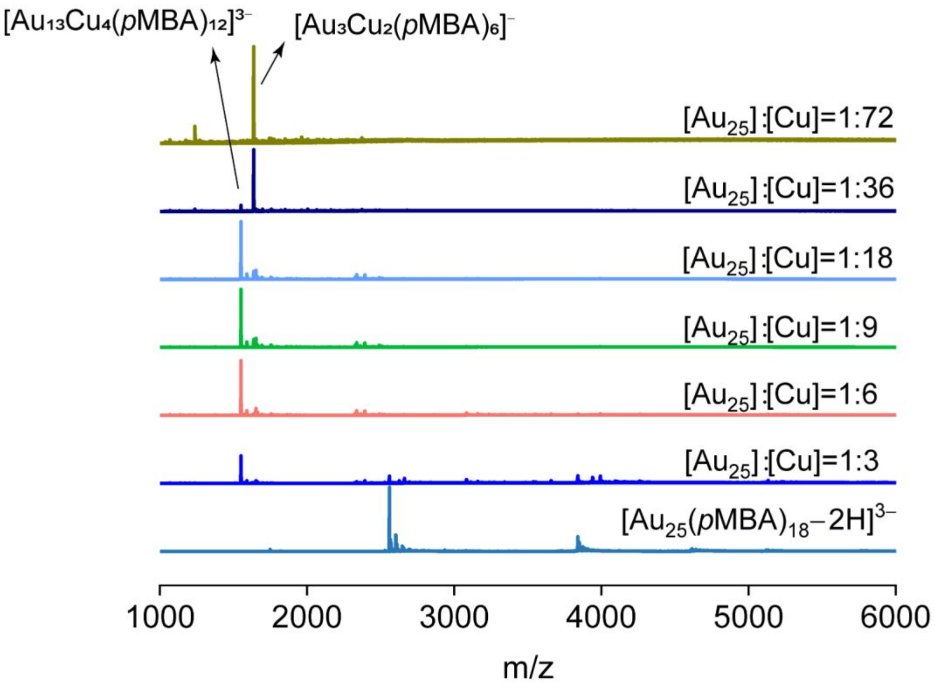

Figure S4. ESI-MS of mixed solution of $[Au_{25}(p\text{MBA})_{18}]^{-}$ NCs with Cu-(*p*MBA) complexes at different ratios of $[Au_{25}]/[Cu]$. The solutions were stirred for 10 min before ESI-MS tests.

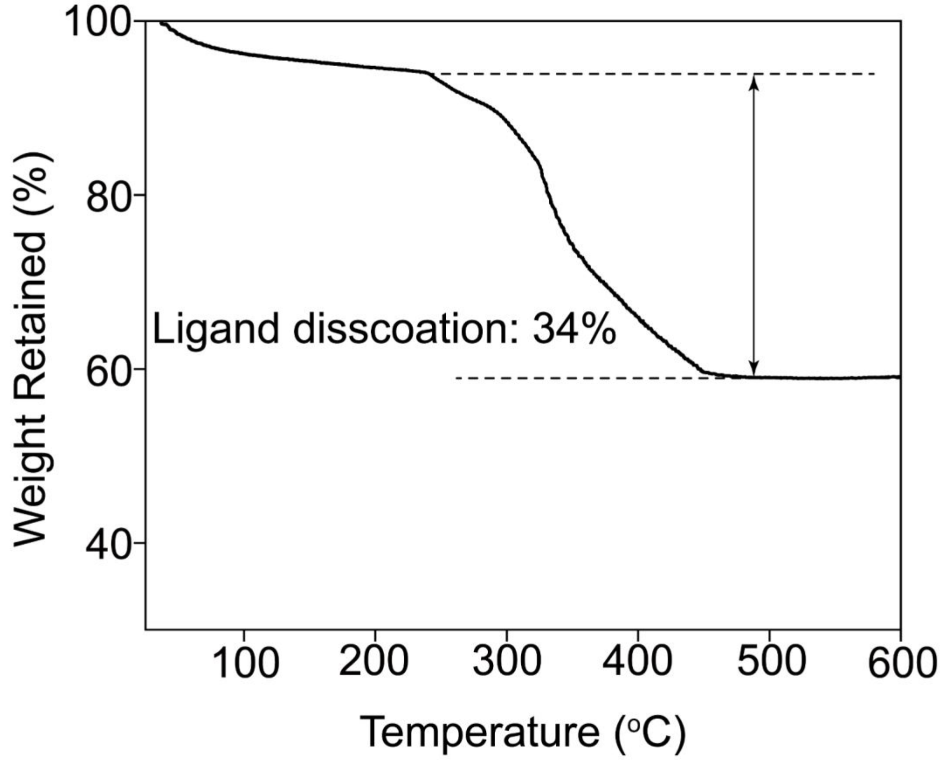

Figure S5. Thermogravimetric analysis of as-synthesized $[Au_{13}Cu_4(p\text{MBA})_{12}]^{3-}$ under air conditions.

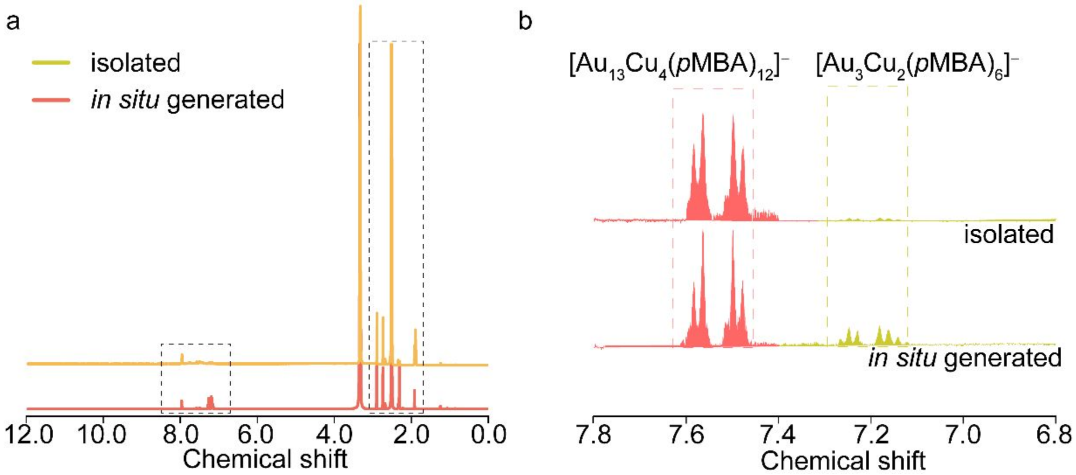

Figure S6. Composition characterization of *in situ* generated and isolated $[Au_{13}Cu_4(p\text{MBA})_{12}]^{3-}$. (a) ^{1}H NMR spectra, (b) a zoom-in view of (a).

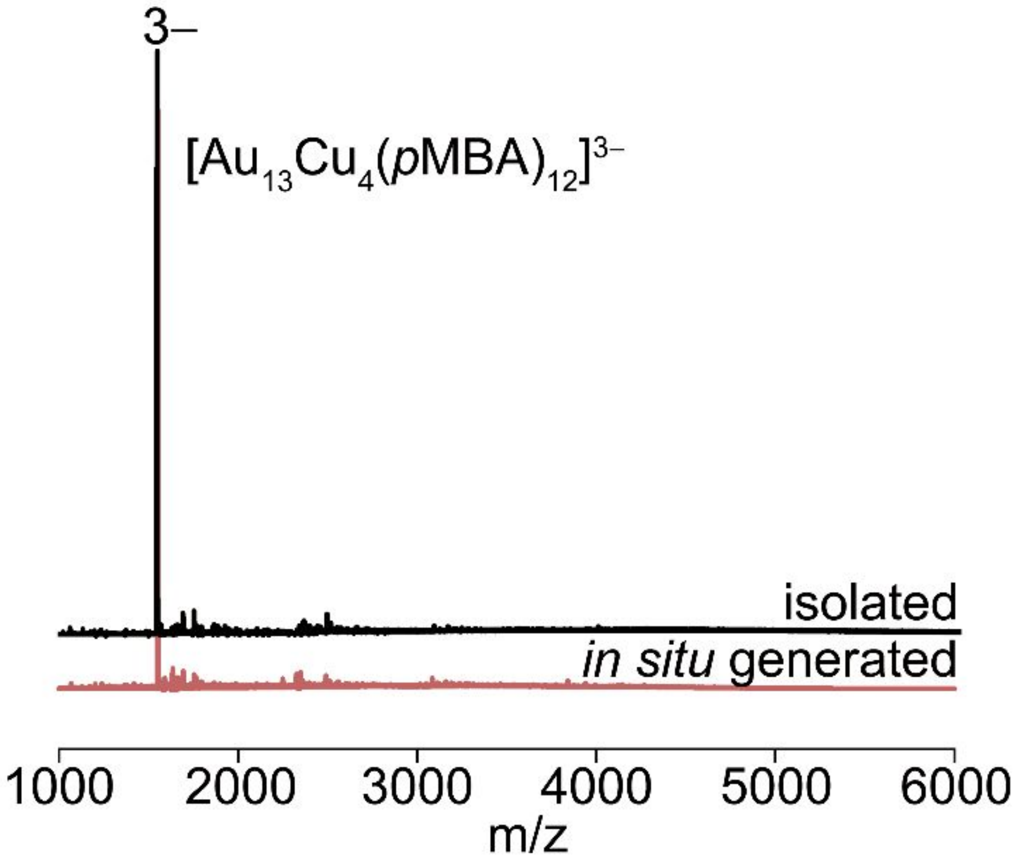

Figure S7. ESI-MS spectra comparison on *in situ* generated and isolated $[Au_{13}Cu_4(p\text{MBA})_{12}]^{3-}$.

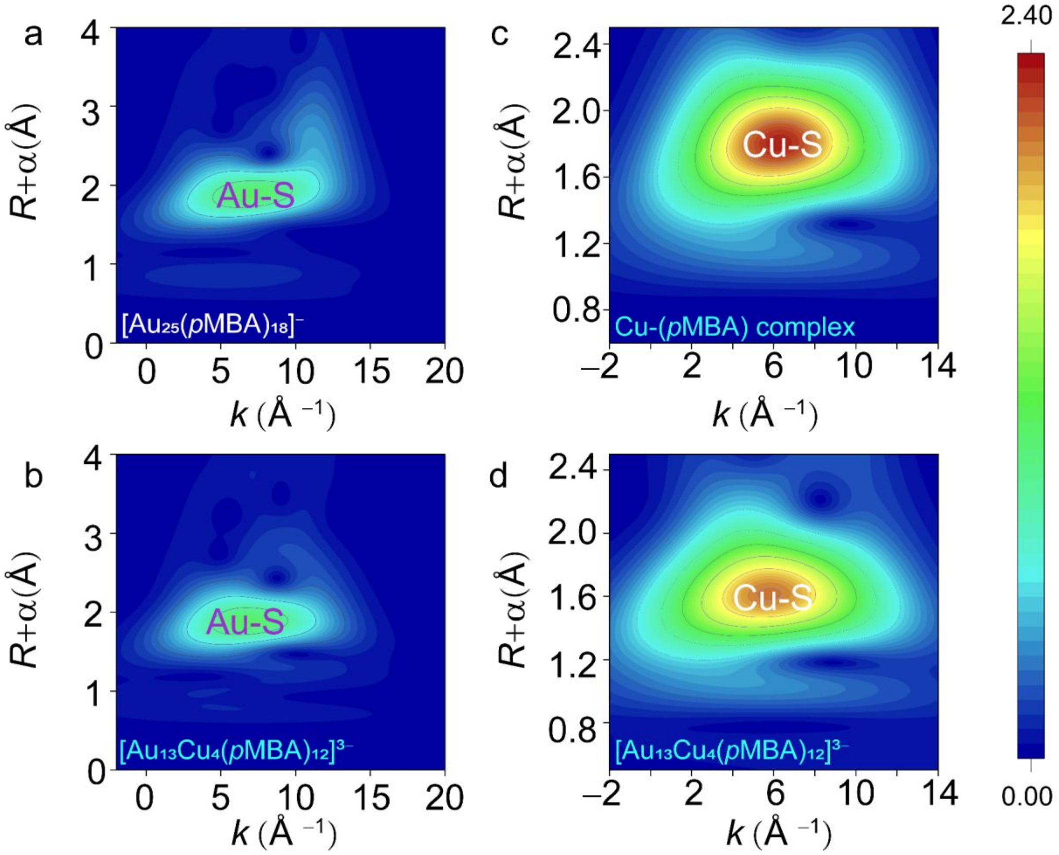

Figure S8. Au L_3 edge and Cu K edge wavelet transform map for k^3-weighted of Cu-(*p*MBA) complex, $[Au_{25}(p\text{MBA})_{18}]^{-}$, and $[Au_{13}Cu_4(p\text{MBA})_{12}]^{3-}$.

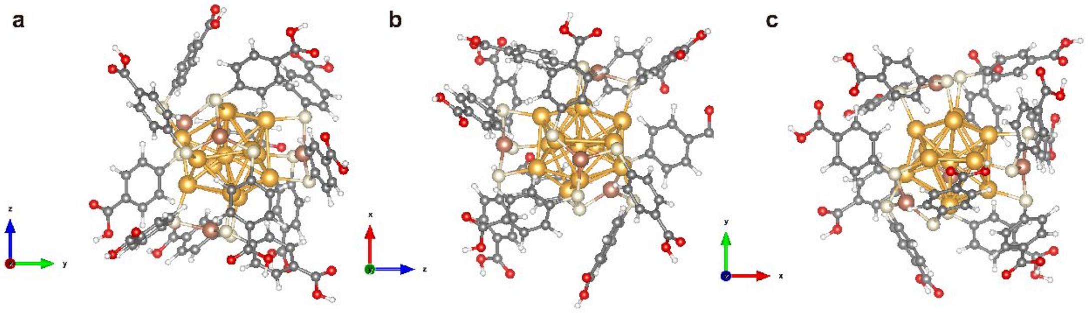

Figure S9. DFT-optimized $[Au_{13}Cu_4(p\text{MBA})_{12}]^{3-}$ structure (yellow = Au, light red = Cu, light yellow = S, red = O, grey = C, white = hydrogen).

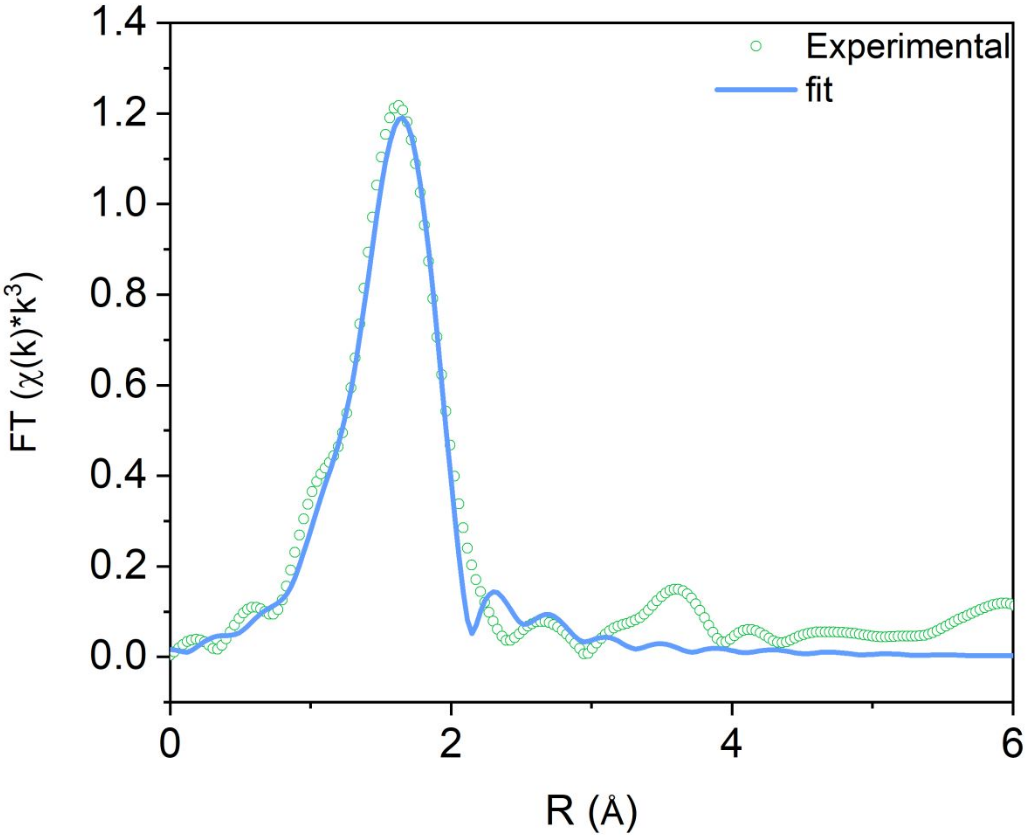

Figure S10. Representative Cu K-edge EXAFS fitting in *R*-space of $[Au_{13}Cu_4(p\text{MBA})_{12}]^{3-}$.

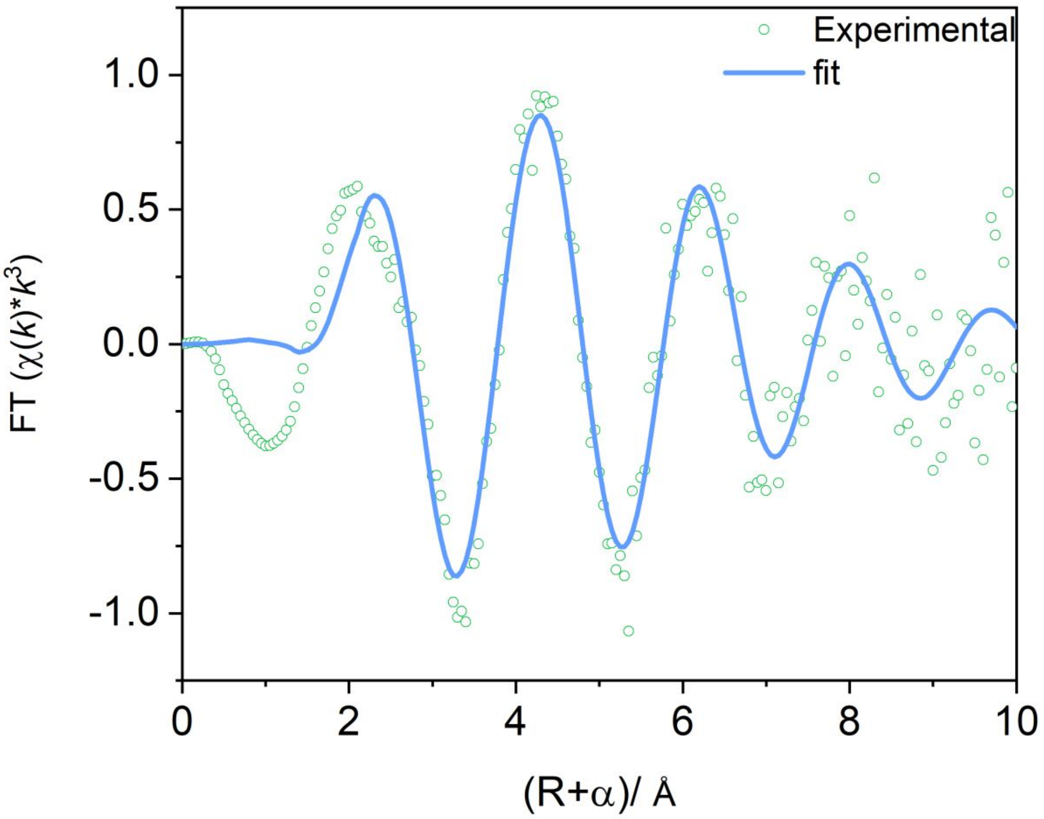

Figure S11. Representative Cu K-edge EXAFS fitting in *k*-space of $[Au_{13}Cu_4(p\text{MBA})_{12}]^{3-}$.

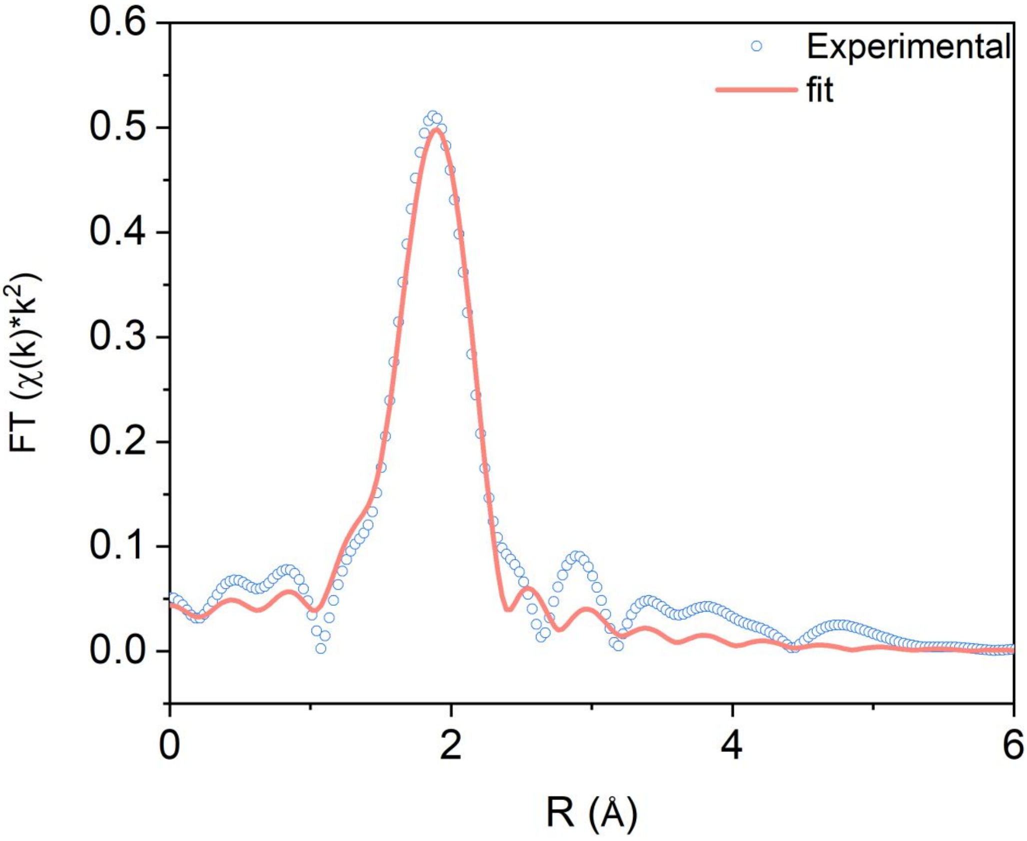

Figure S12. Representative Au L_3-edge EXAFS fitting in *R*-space of $[Au_{13}Cu_4(p\text{MBA})_{12}]^{3-}$.

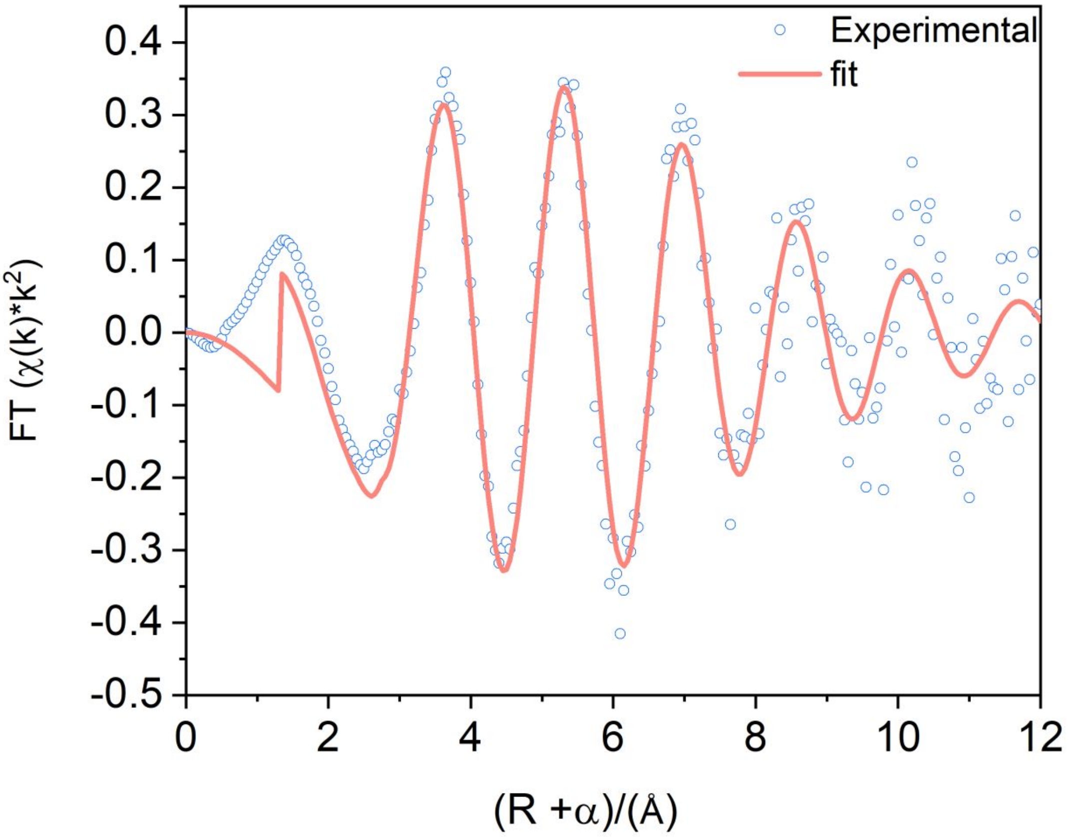

Figure S13. Representative Au L_3-edge EXAFS fitting in *k*-space of $[Au_{13}Cu_4(p\text{MBA})_{12}]^{3-}$.

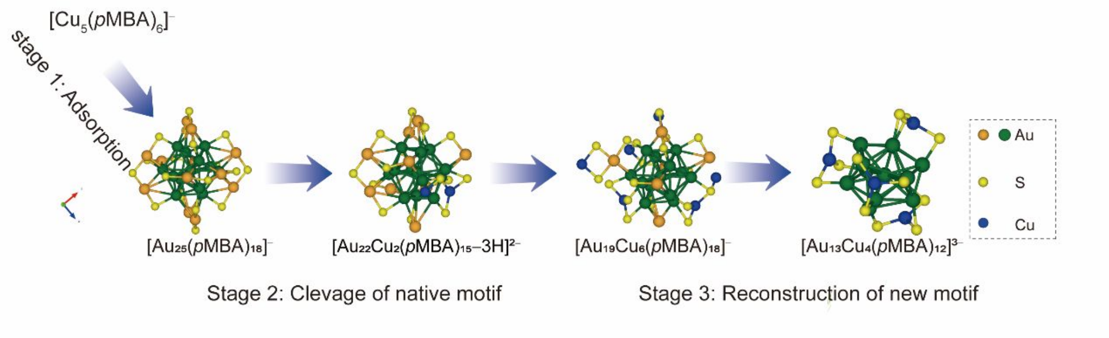

Figure S14. Schematic illustration of motif editing process.

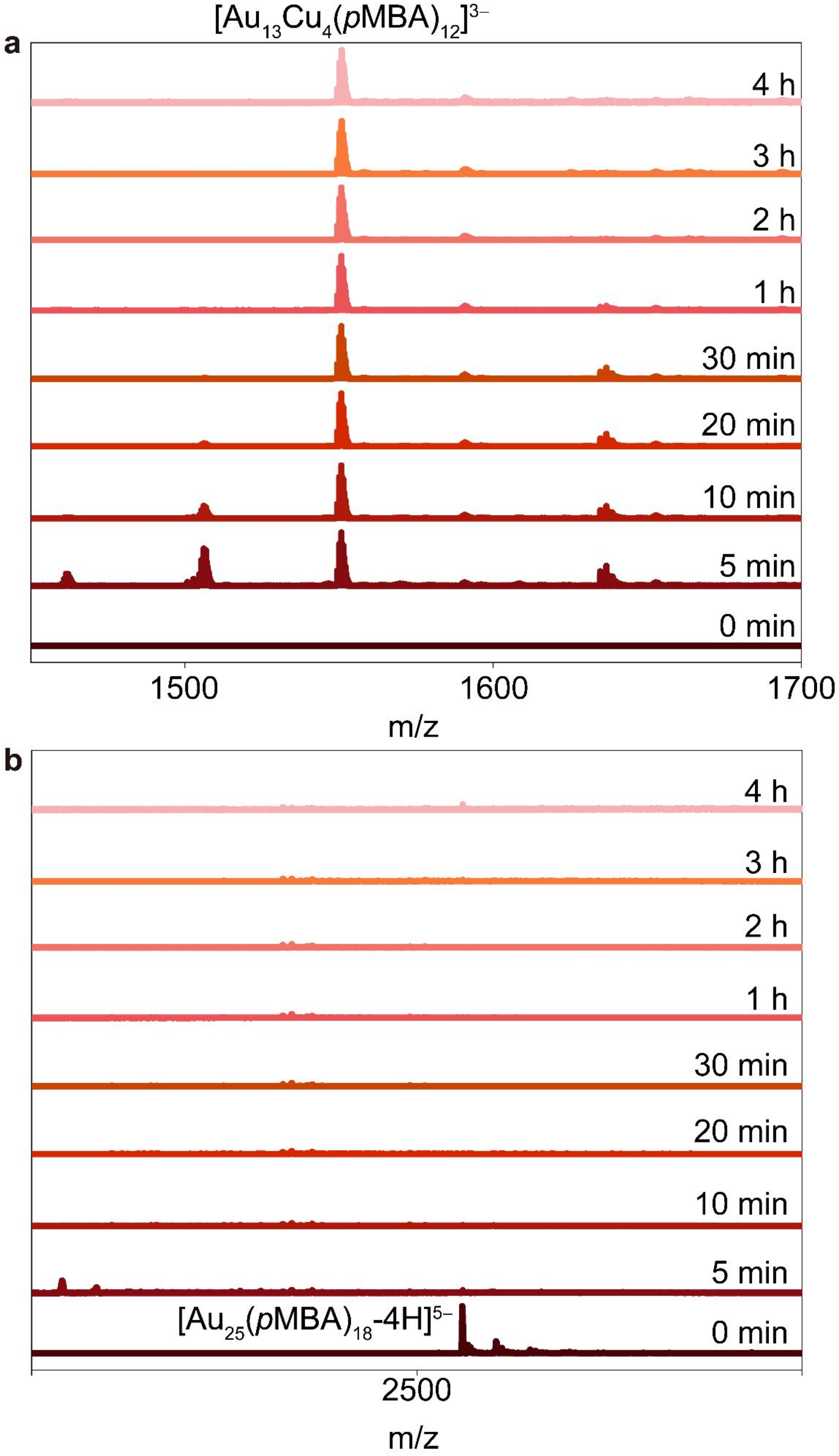

[Au13Cu4(pMBA)12]3−
a
4 h
3 h
2 h
1 h
30 min
20 min
10 min
5 min
0 min
1500
1600
1700
m/z
b
4 h
3 h
2 h
1 h
30 min
20 min
10 min
5 min
[Au25(pMBA)18-4H]5−
0 min
2500
m/z

Figure S15. Zoom-in ESI-MS of Figure 3b. a, low region and b, high region. The immediate emergence of $[Au_{13}Cu_4(p\text{MBA})_{12}]^{3-}$ accompanied by transient intermediates such as $[Au_{13}Cu_7(p\text{MBA})_9\text{-}2H]^{3-}$ (*m/z* = 1460), $[Au_{14}Cu_6(p\text{MBA})_9]^{3-}$ (*m/z* = 1506.1), and $[Au_3Cu_2(p\text{MBA})_6]^{-}$ (*m/z* = 1636.7). These species dominate after 1 hour but eventually converge toward a compositionally focused final product: $[Au_{13}Cu_4(p\text{MBA})_{12}]^{3-}$, with minor residual amounts of $[Au_3Cu_2(p\text{MBA})_6]^{-}$ persisting beyond 4 hours.

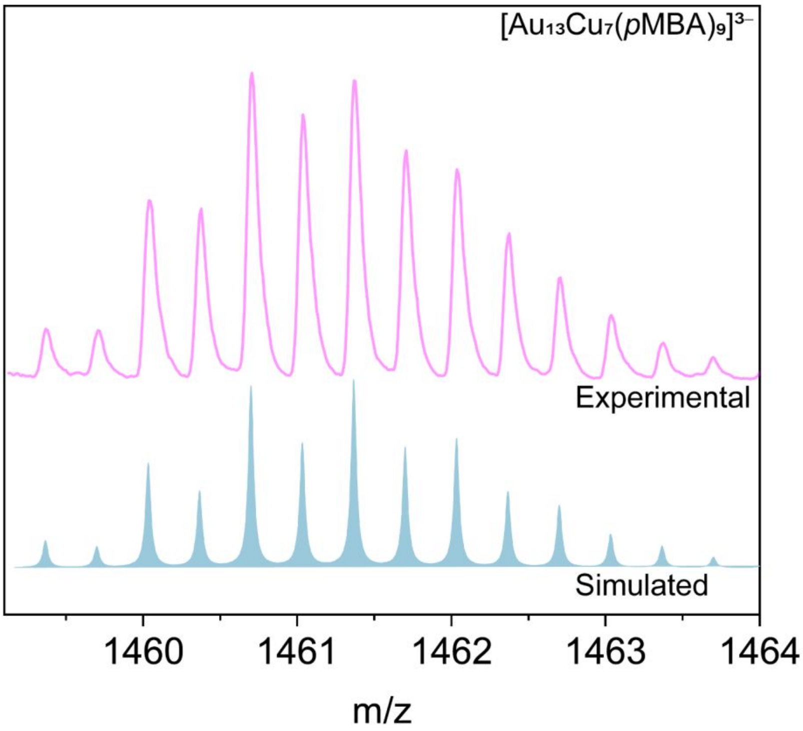

Figure S16. ESI-MS spectra of $[Au_{13}Cu_7(p\mathrm{MBA})_9]^{3-}$.

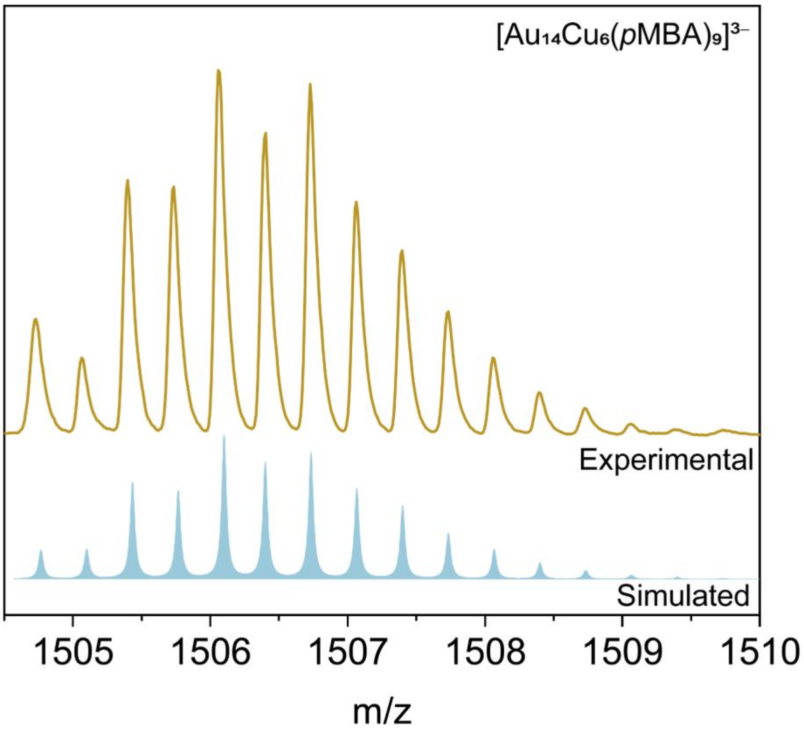

Figure S17. ESI-MS spectra of $[Au_{14}Cu_6(p\text{MBA})_9]^{3-}$.

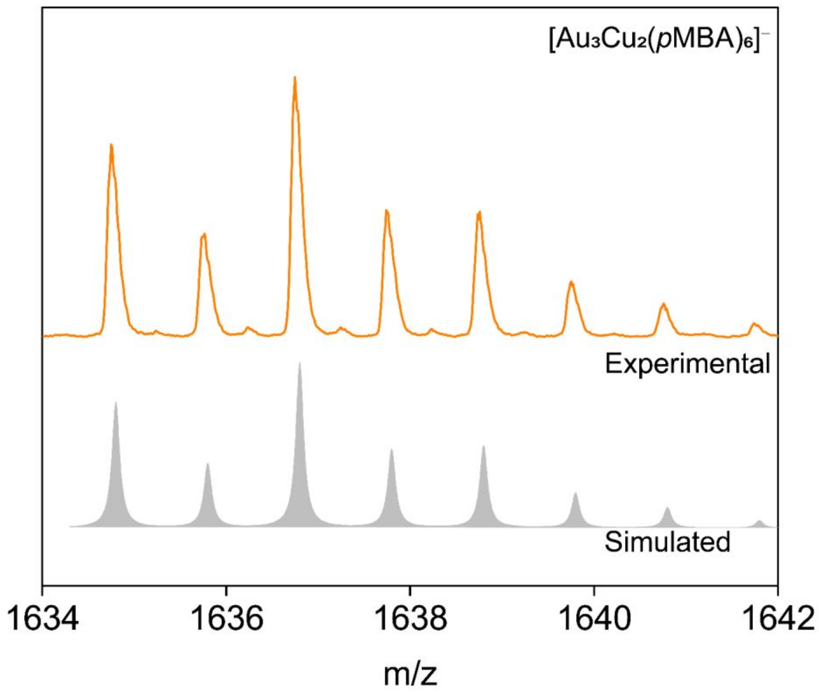

Figure S18. ESI-MS of $[Au_3Cu_2(p\text{MBA})_6]^-$.

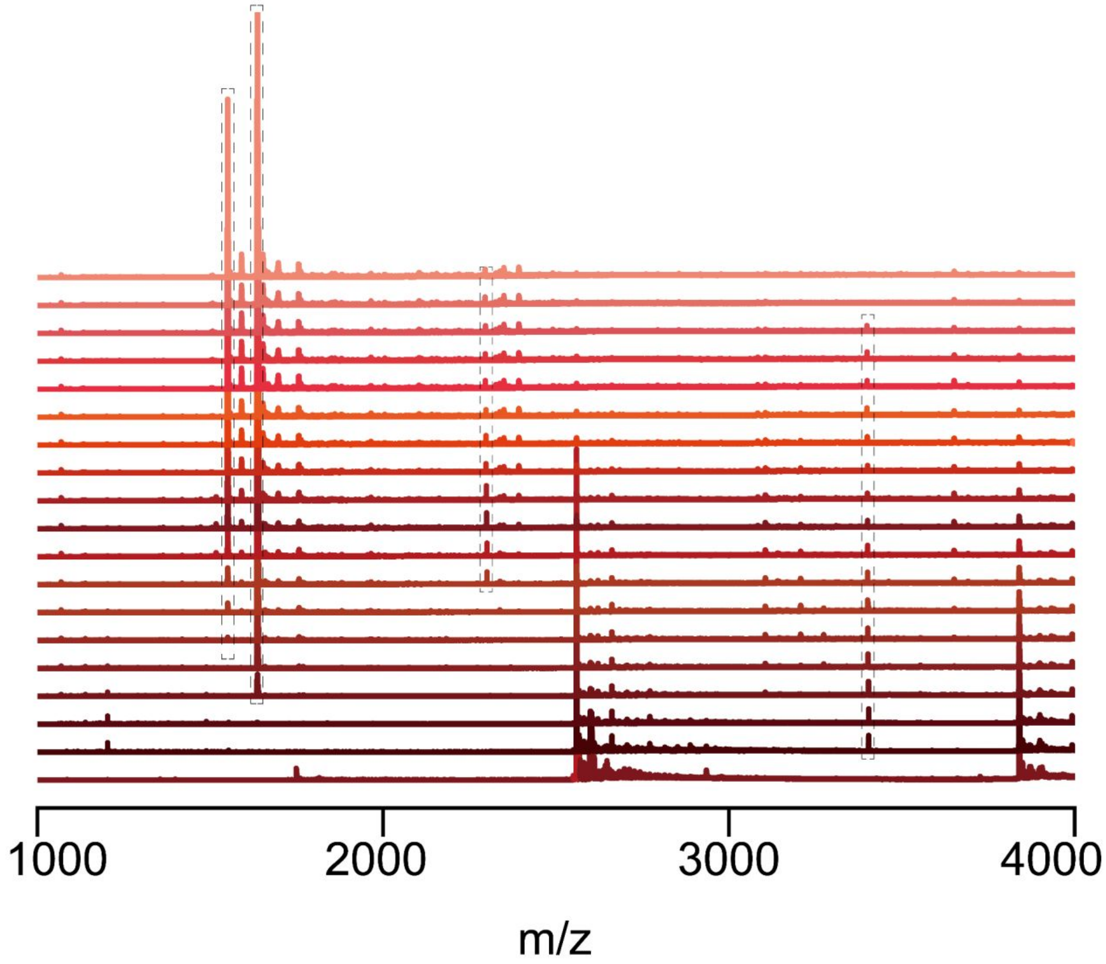

Figure S19. ESI-MS analysis uncovers the formation of distinct intermediates, and corresponding peak intensity profiles of precursors and intermediates.

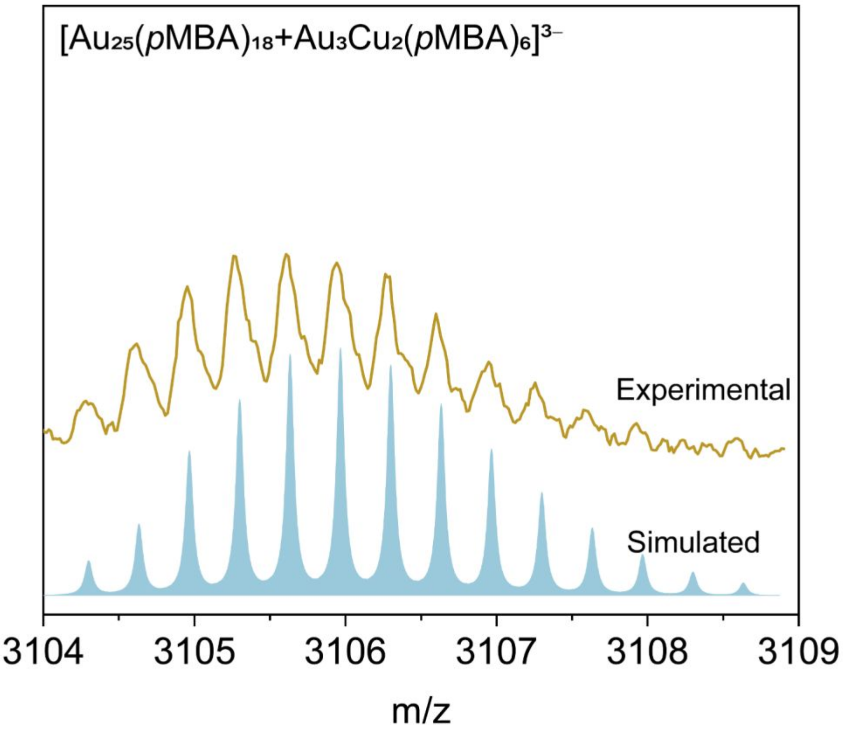

Figure S20. ESI-MS of $[Au_{25}(p\text{MBA})_{18}+ Au_3Cu_2(p\text{MBA})_6]^{3-}$.

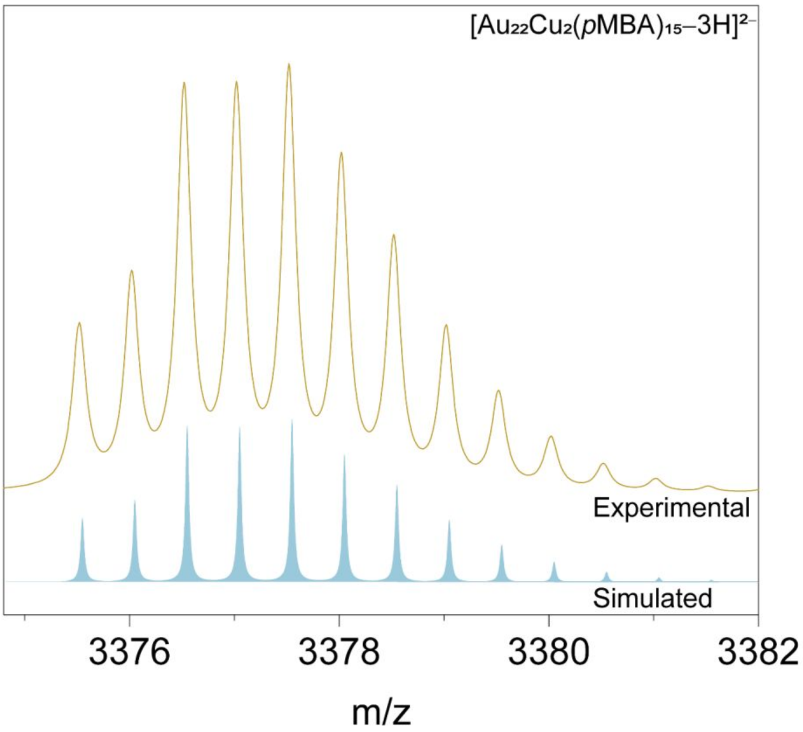

Figure S21. ESI-MS of $[Au_{22}Cu_2(p\text{MBA})_{15}\text{–}3H]^{2-}$.

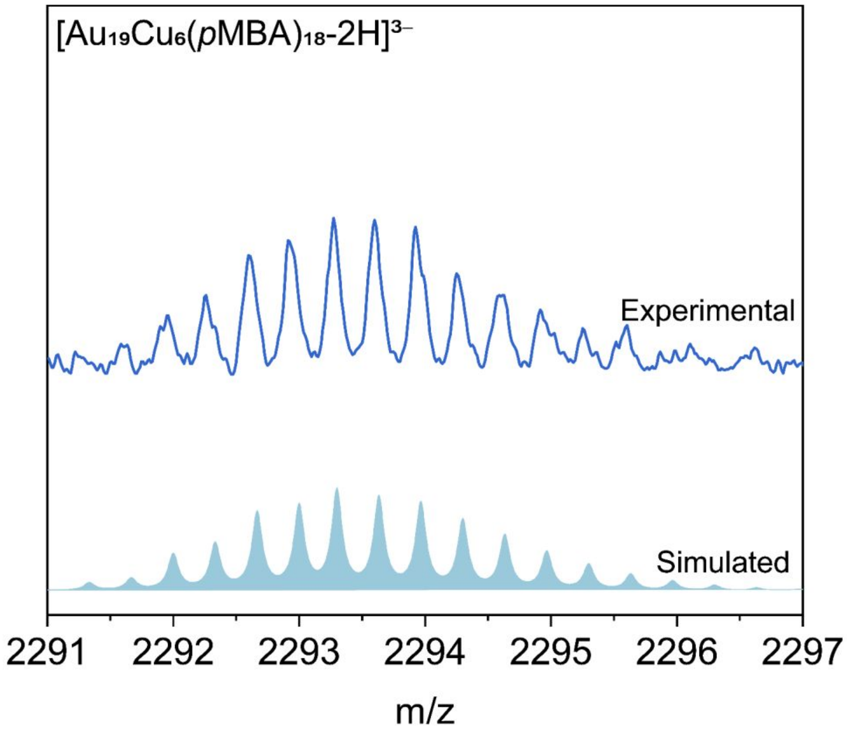

Figure S22. ESI-MS of $[Au_{19}Cu_6(p\text{MBA})_{18}]^-$.

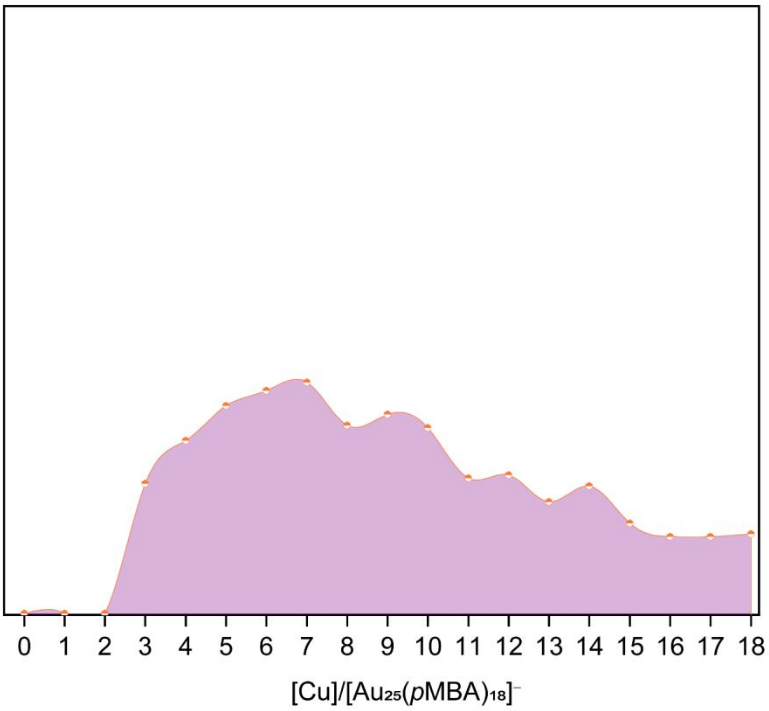

Figure S23. The intensity evolution of [Au_{25}(*p*MBA)$_{18}$+ Au_3Cu_2(*p*MBA)$_6$]$^{3-}$ during the stepwise reaction between [Au_{25}(*p*MBA)$_{18}$]$^{-}$ and Cu-(*p*MBA) complex.

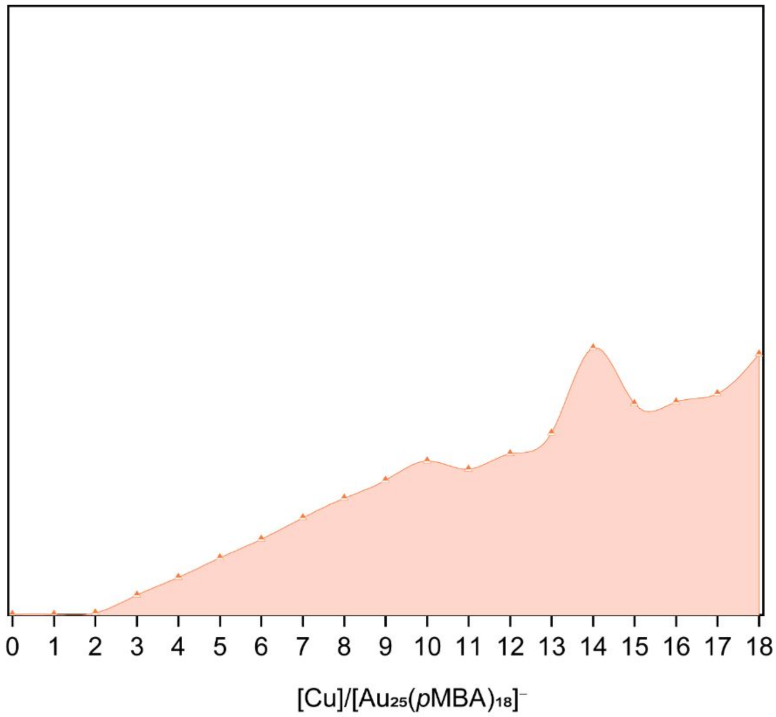

Figure S24. The intensity evolution of $[Au_3Cu_2(p\text{MBA})_6]^-$ during the stepwise reaction between $[Au_{25}(p\text{MBA})_{18}]^-$ and Cu-(*p*MBA) complexes.

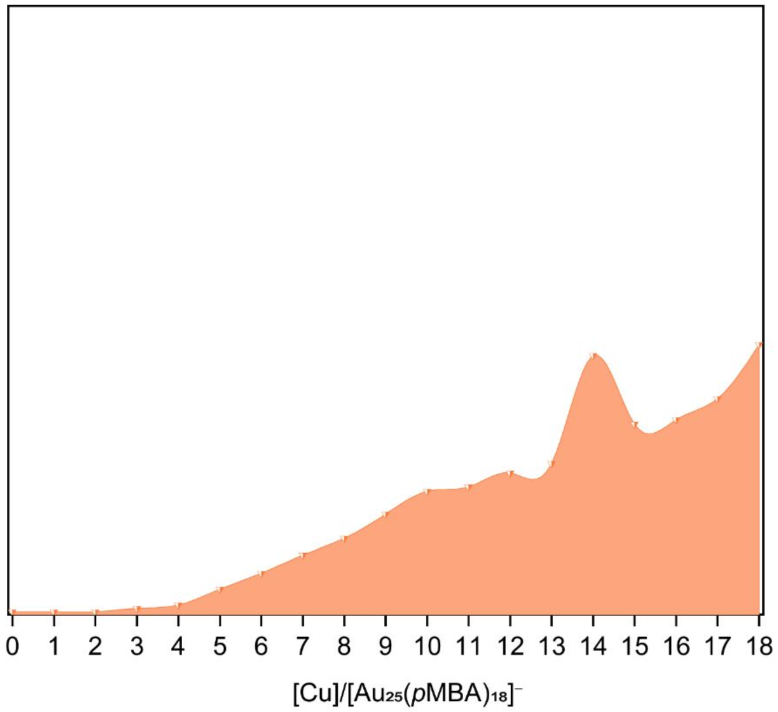

Figure S25. The intensity evolution of $[Au_6Cu_4(p\mathrm{MBA})_{12}]^-$ during the stepwise reaction between $[Au_{25}(p\mathrm{MBA})_{18}]^-$ and Cu-(*p*MBA) complex.

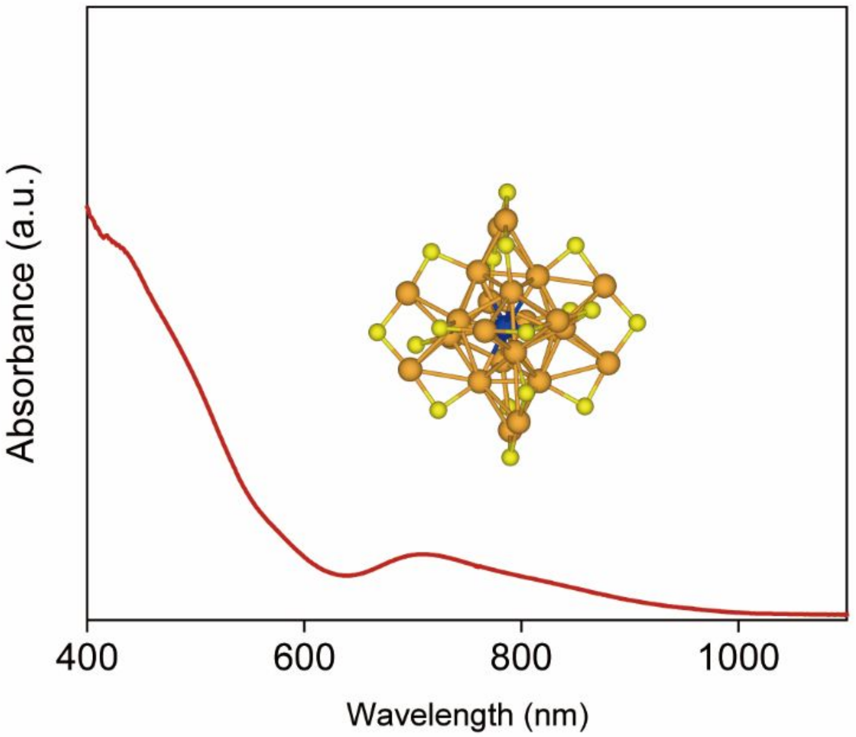

Figure S26. UV-vis absorption spectra of AuCu-alloy NCs synthesized by co-reduction method.(inset: proposed structure of $[Au_{24}Cu(p\mathrm{MBA})_{18}]^{-}$ color code: green = Au, blue = Cu, yellow = S)

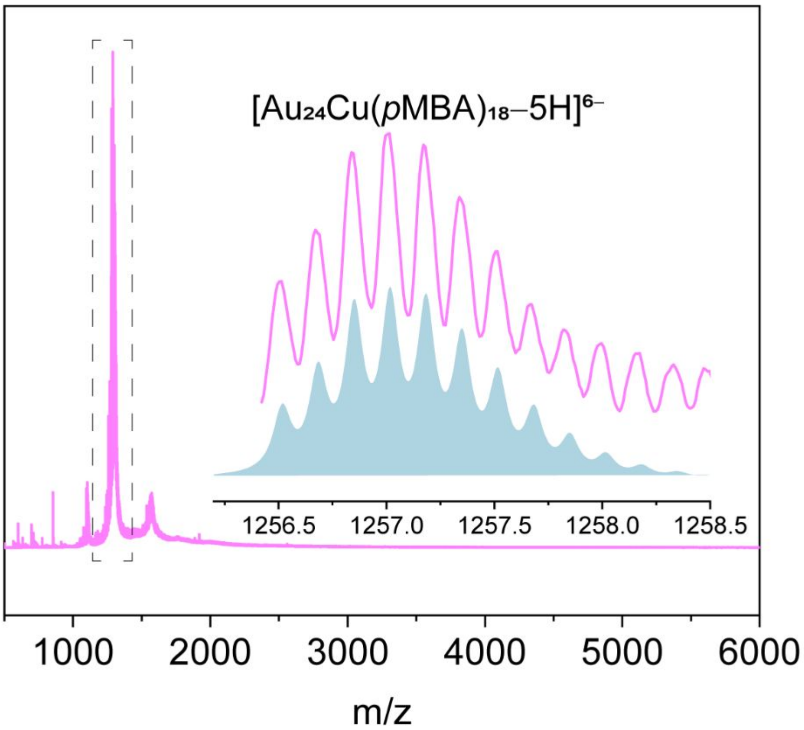

Figure S27. ESI-MS spectra of AuCu-alloy NCs synthesized by co-reduction method. The insert shows the zoomed-in view of mass spectra within the dashed rectangle (the filled peaks in the inset belong to the simulated isotope distribution peaks).

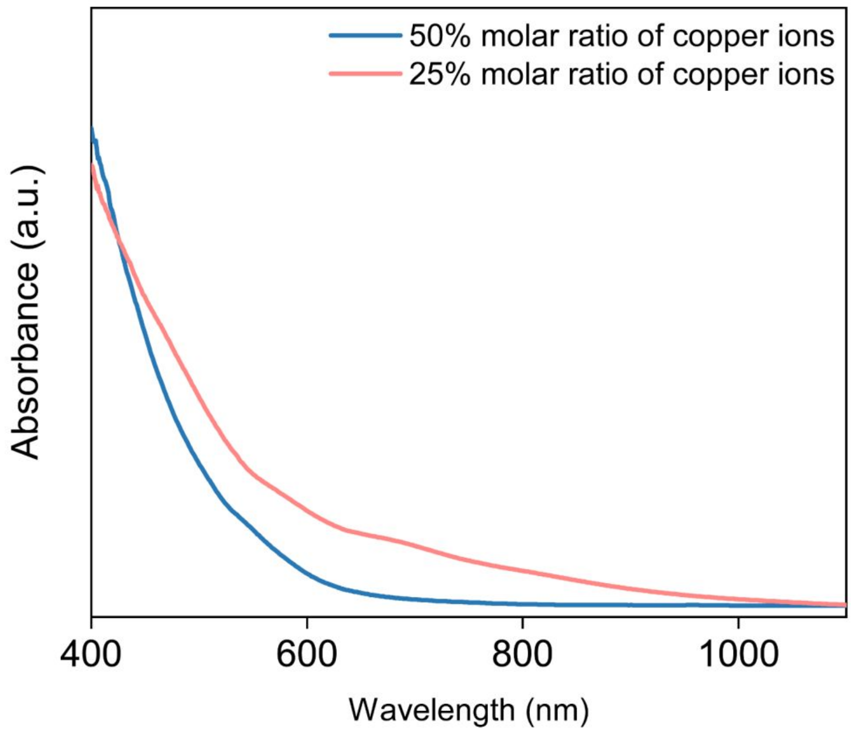

Figure S28. UV-vis absorption spectra of copper doped $[Au_{25}(p\text{MBA})_{18}]^{-}$ NCs with different feeding molar ratio of copper ions.

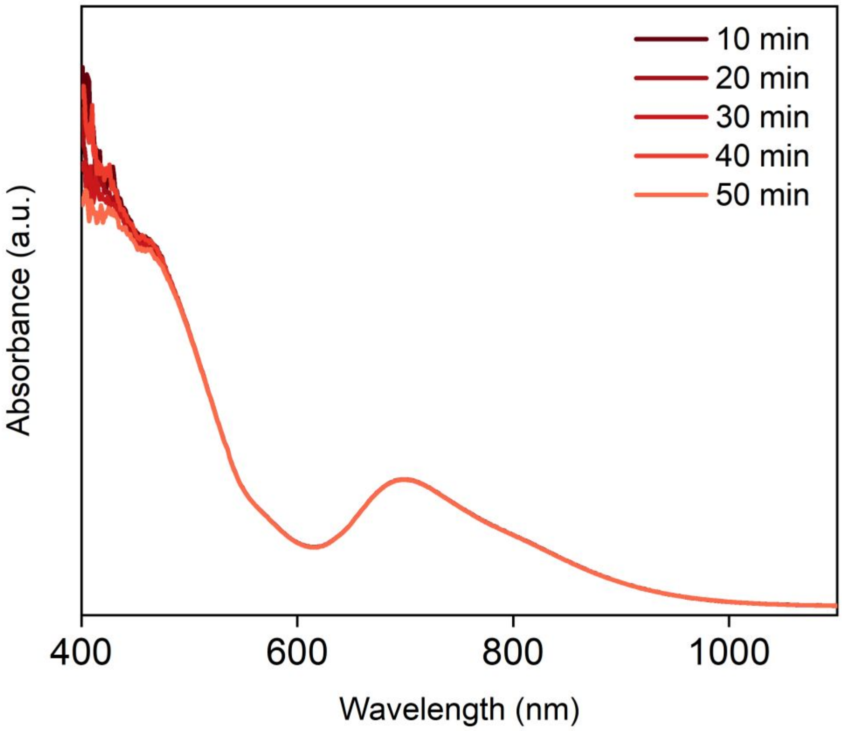

Figure S29. UV-vis absorption spectra of mixed solution of $[Au_{25}(p\text{MBA})_{18}]^-$ NCs with Cu(II) ions.

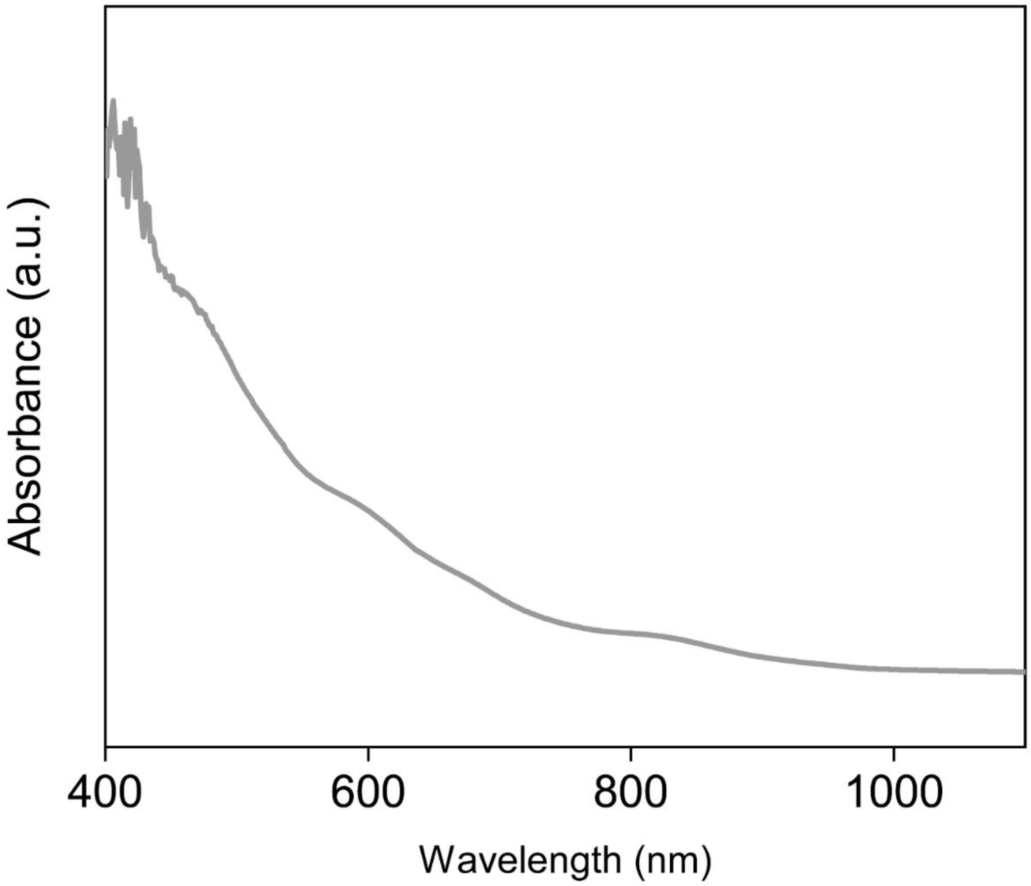

Figure S30. UV-vis absorption spectra of $[Au_{38}(p\mathrm{MBA})_{26}]^0$ NCs.

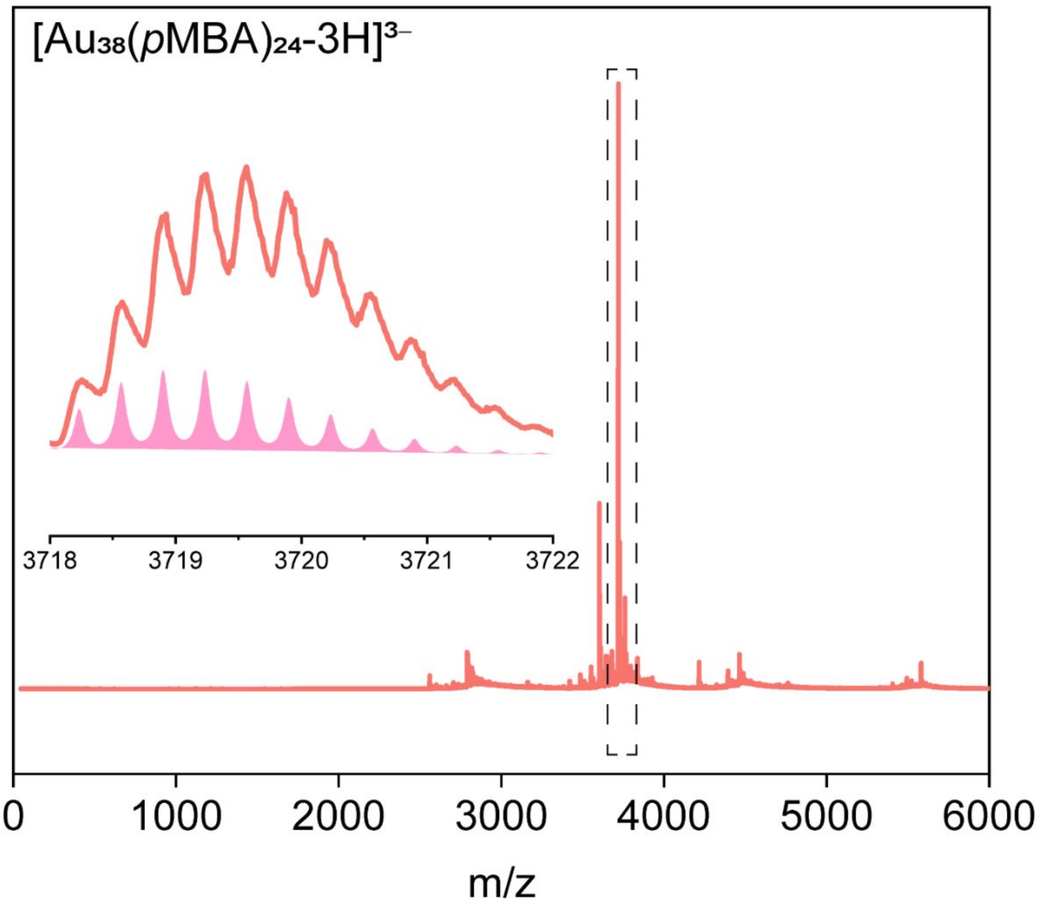

Figure S31. ESI-MS spectra of $[Au_{38}(p\text{MBA})_{26}]^0$ NCs. The insert shows the zoomed-in view of mass spectra within the dashed rectangle (the filled peaks in the inset belong to the simulated isotope distribution peaks).

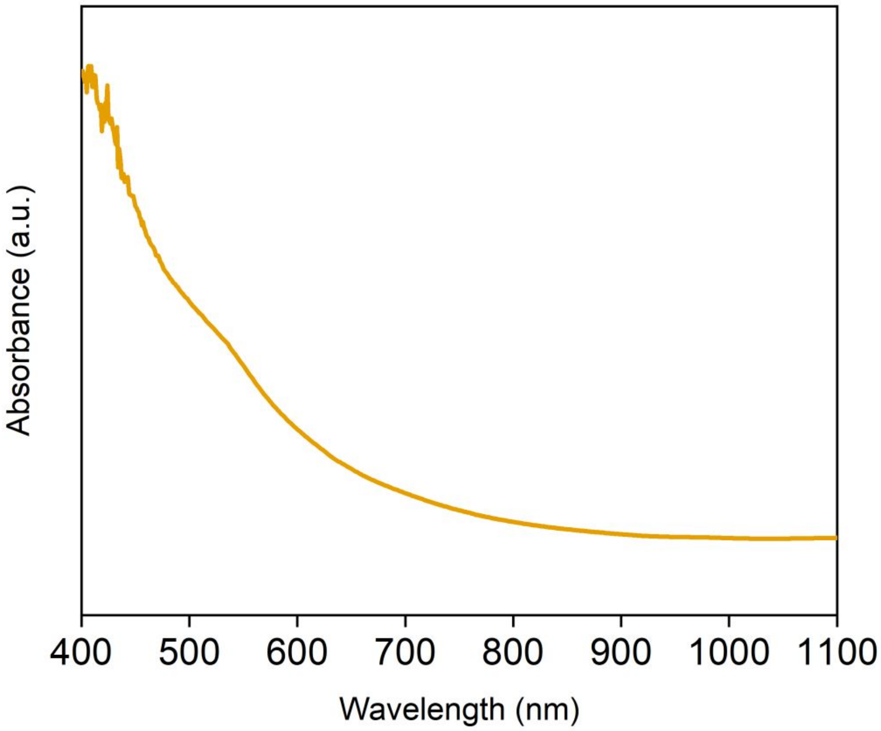

Figure S32. UV-vis absorption spectra of mixed solution of $[Au_{38}(p\mathrm{MBA})_{26}]^0$ NCs with Cu-(*p*MBA) complexes with a feeding ratio of $[Cu]/[Au_{38}]$ = 10%.

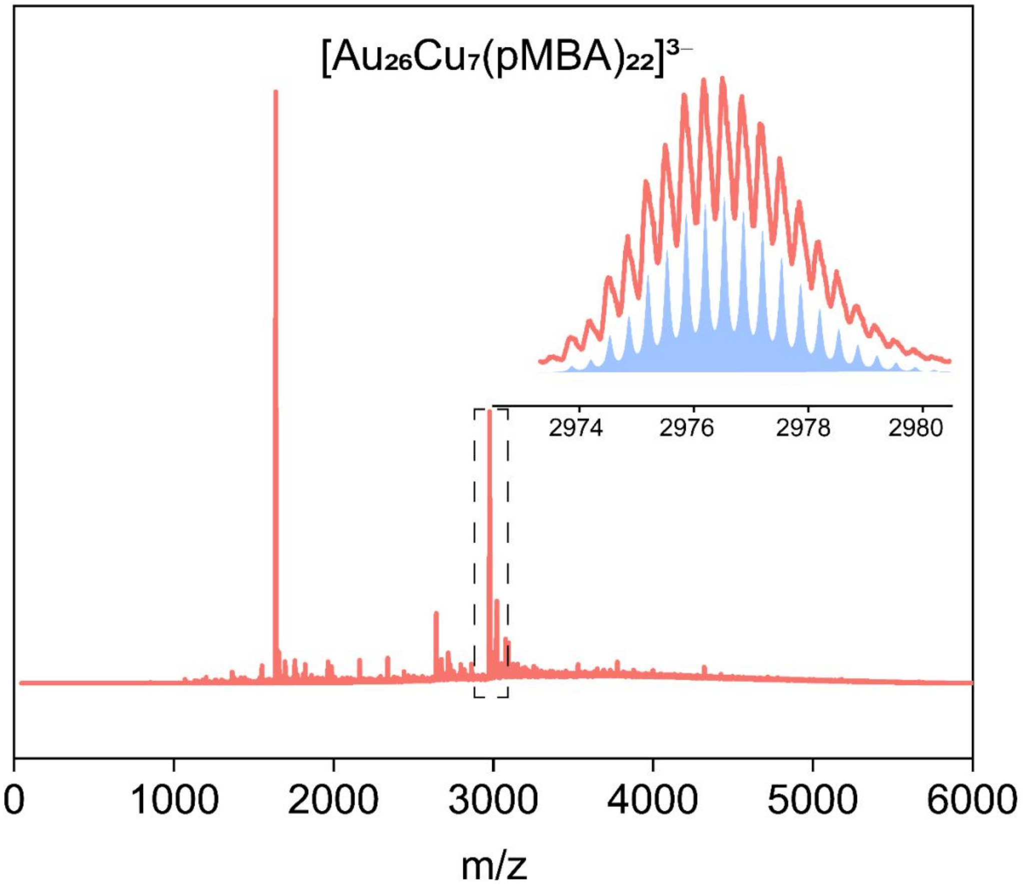

Figure S33. ESI-MS of mixed solution of $[Au_{38}(pMBA)_{26}]^0$ NCs with Cu-(*p*MBA) complexes with a feeding $[Cu]/[Au_{38}(SR)_{24}]$ ratio of 10%. The insert shows the zoomed-in view of mass spectra within the dashed rectangle (the filled peaks in the inset belong to the simulated isotope distribution peaks).

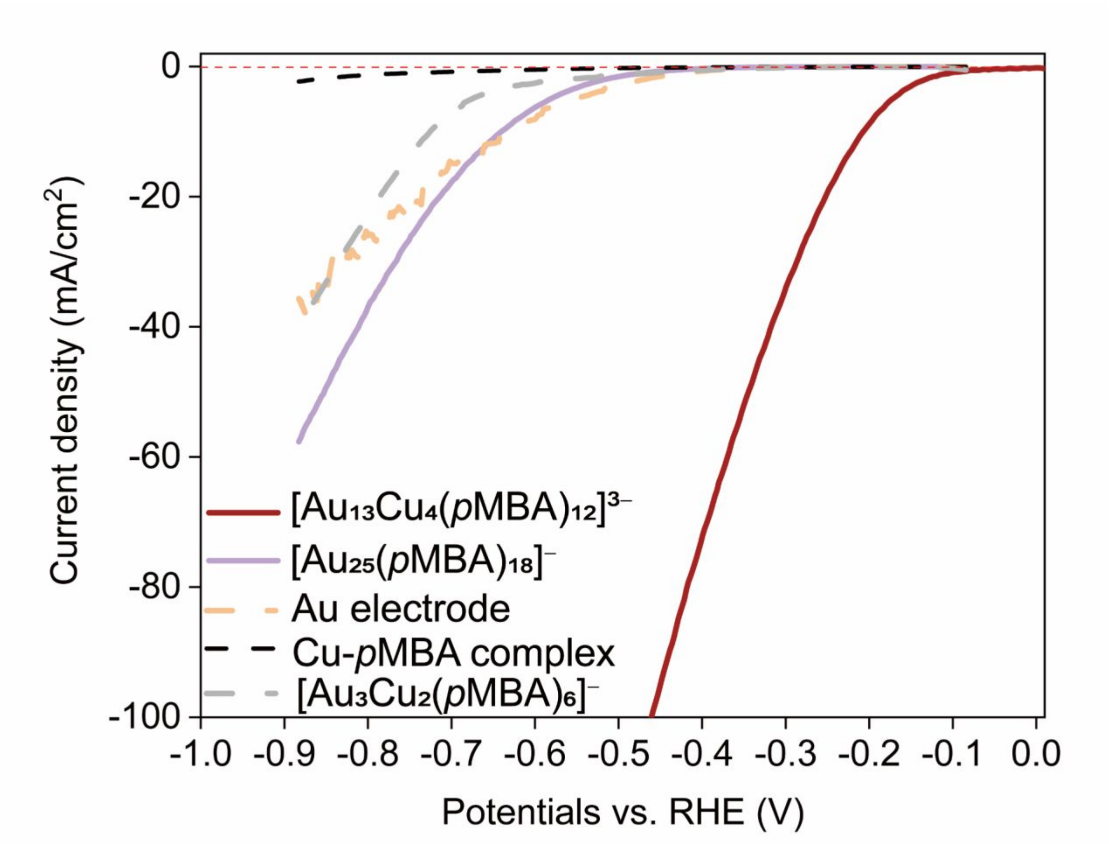

Figure S34. Linear sweep voltammetry (LSV) of $[Au_{25}(p\text{MBA})_{18}]^{-}$ and $[Au_{13}Cu_4(p\text{MBA})_{12}]^{3-}$ NCs.

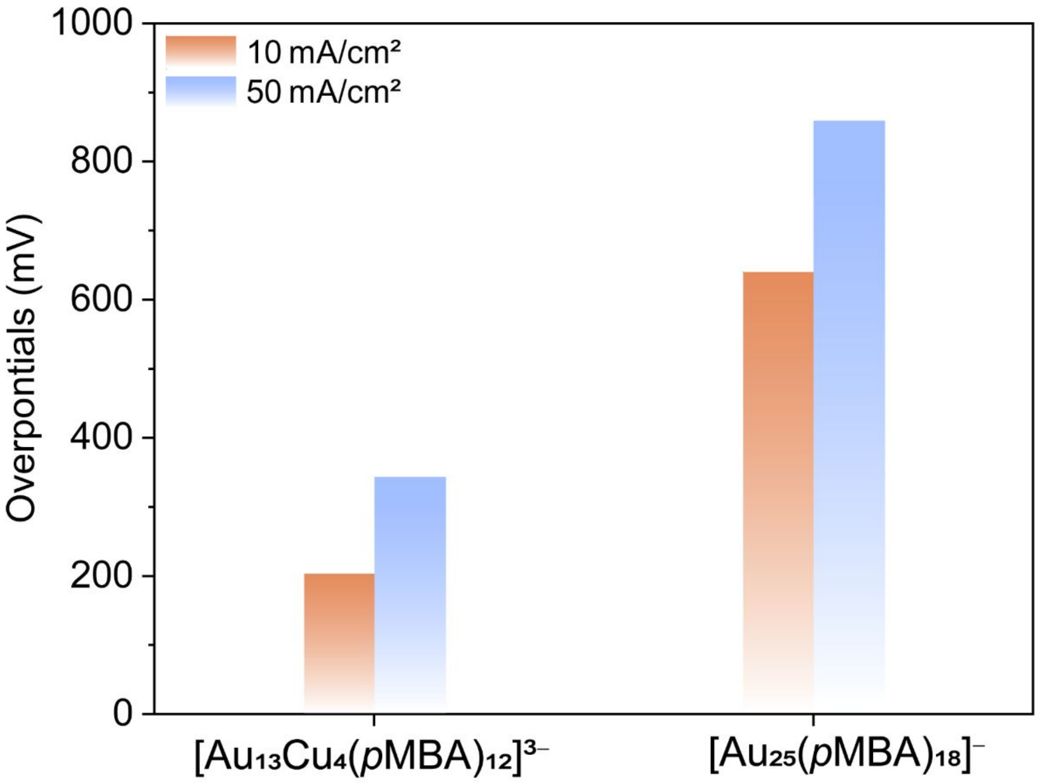

Figure S35. Overpotentials at 10 and 50 mA cm^{-2}.

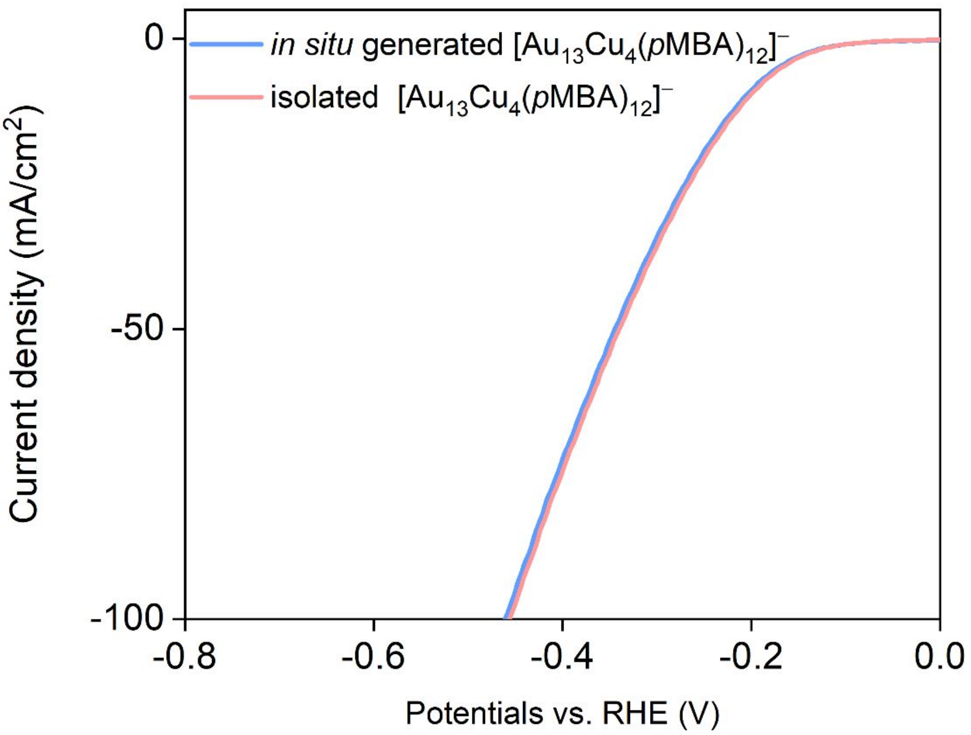

Figure S36. Linear sweep voltammetry curve comparison of *in situ* generated and isolated $[Au_{13}Cu_4(pMBA)_{12}]^{3-}$. No significant difference was observed, indicating that the side product $[Au_3Cu_2(pMBA)_6]^-$ made negligible contribution to HER performance.

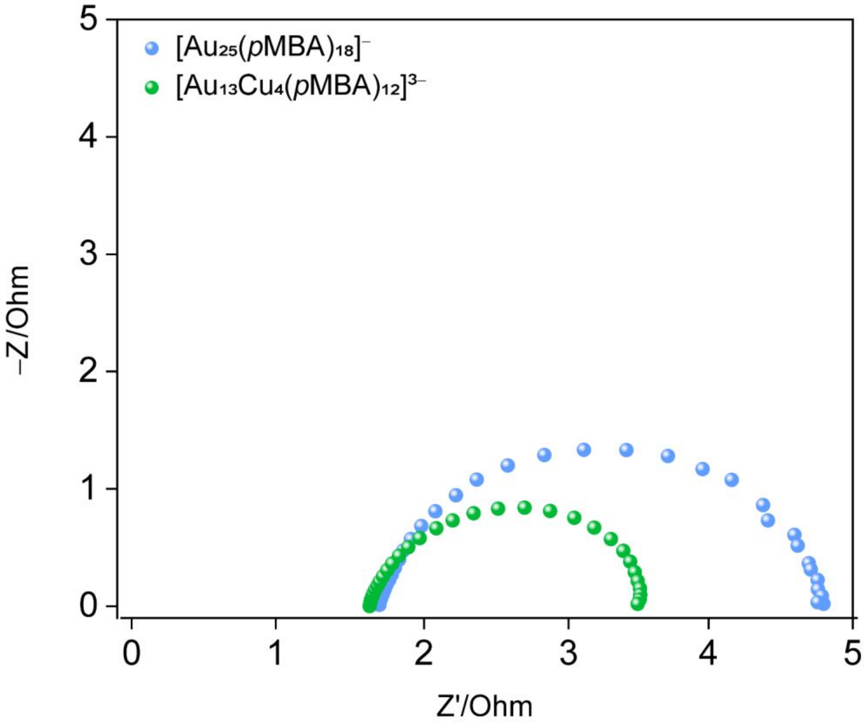

Figure S37. Electrochemical impedance spectra of $[Au_{13}Cu_4(p\text{MBA})_{12}]^{3-}$ and $[Au_{25}(p\text{MBA})_{18}]^{-}$.

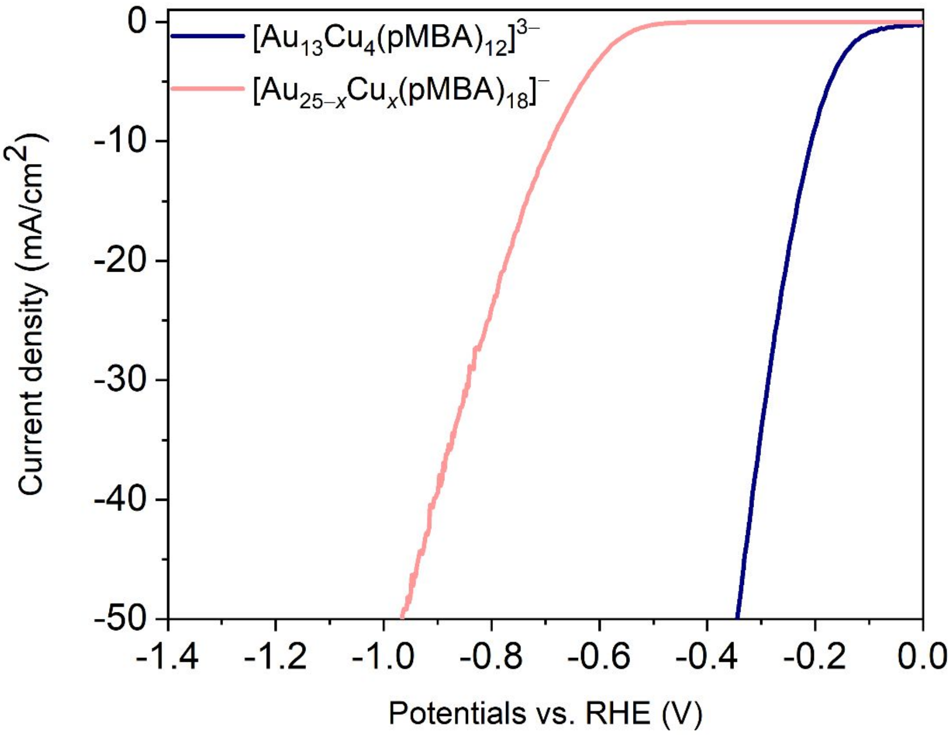

Figure S38. LSV of $[Au_{13}Cu_4(p\text{MBA})_{12}]^{3-}$ NCs and $[Au_{25\text{-}x}Cu_x(p\text{MBA})_{18}]^{-}$ NCs (x = 0, 1).

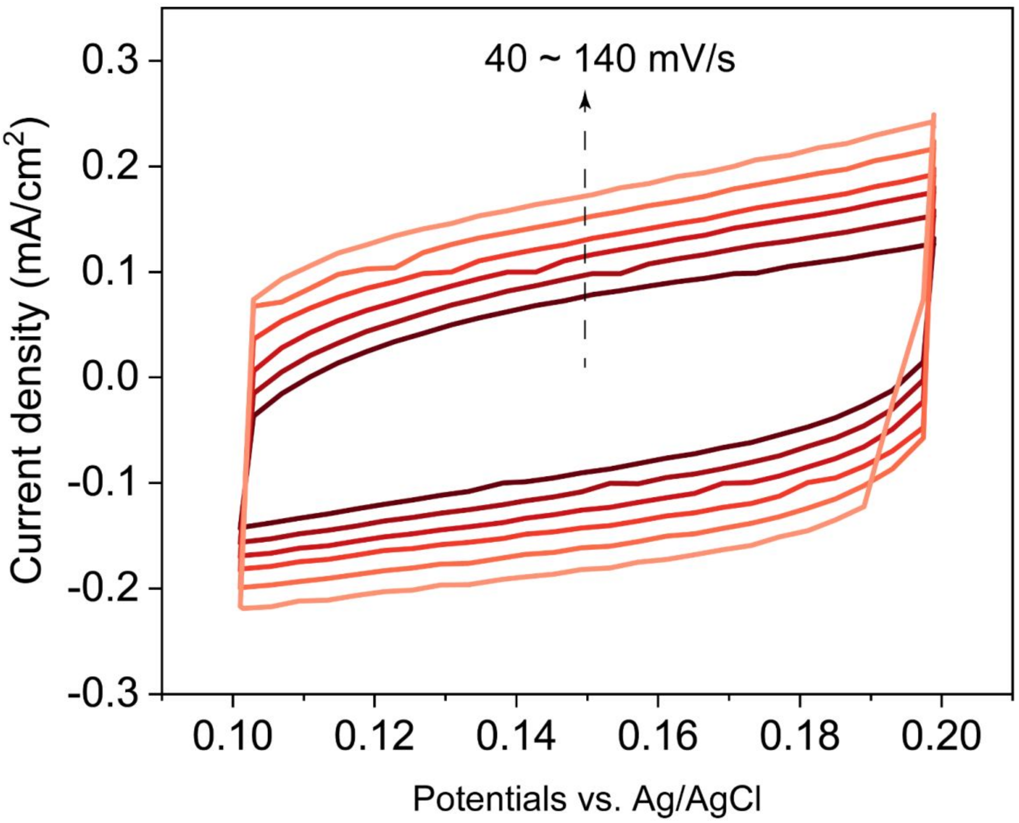

Figure S39. Cyclic voltammetry (CV) curves employed to determine the electrochemical surface area (ECSA) of $[Au_{13}Cu_4(p\text{MBA})_{12}]^{3-}$ NCs. CV curves were recorded over a range of scan rates, specifically from 40 to 140 mV s^{-1}, with an interval of 20 mV s^{-1}. This range of scan rates allows for a reliable assessment of the capacitive current response, which is then used to calculate the ECSA of the nanoclusters.

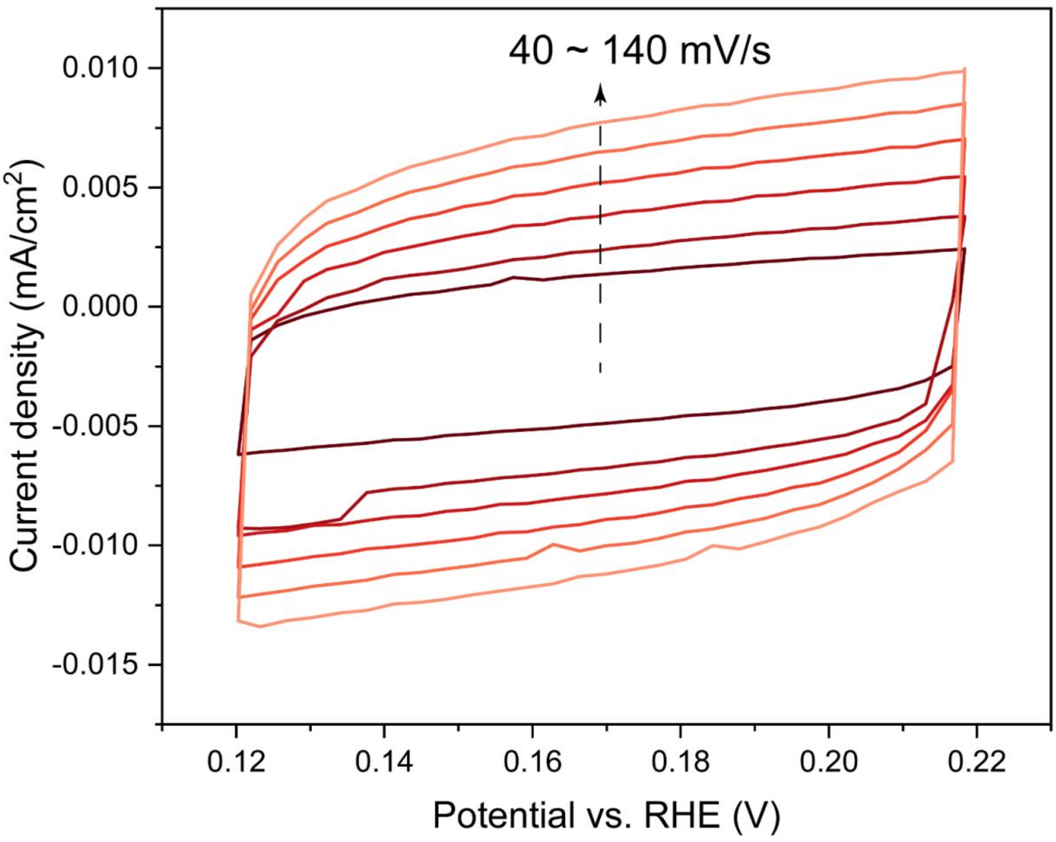

Figure S40. CV curves employed to determine the ECSA of $[Au_{25}(p\mathrm{MBA})_{18}]^-$ NCs. CV curves were recorded over a range of scan rates, specifically from 40 to 140 mV s^{-1}, with an interval of 20 mV/s. This range of scan rates allows for a reliable assessment of the capacitive current response, which is then used to calculate the ECSA of the nanoclusters.

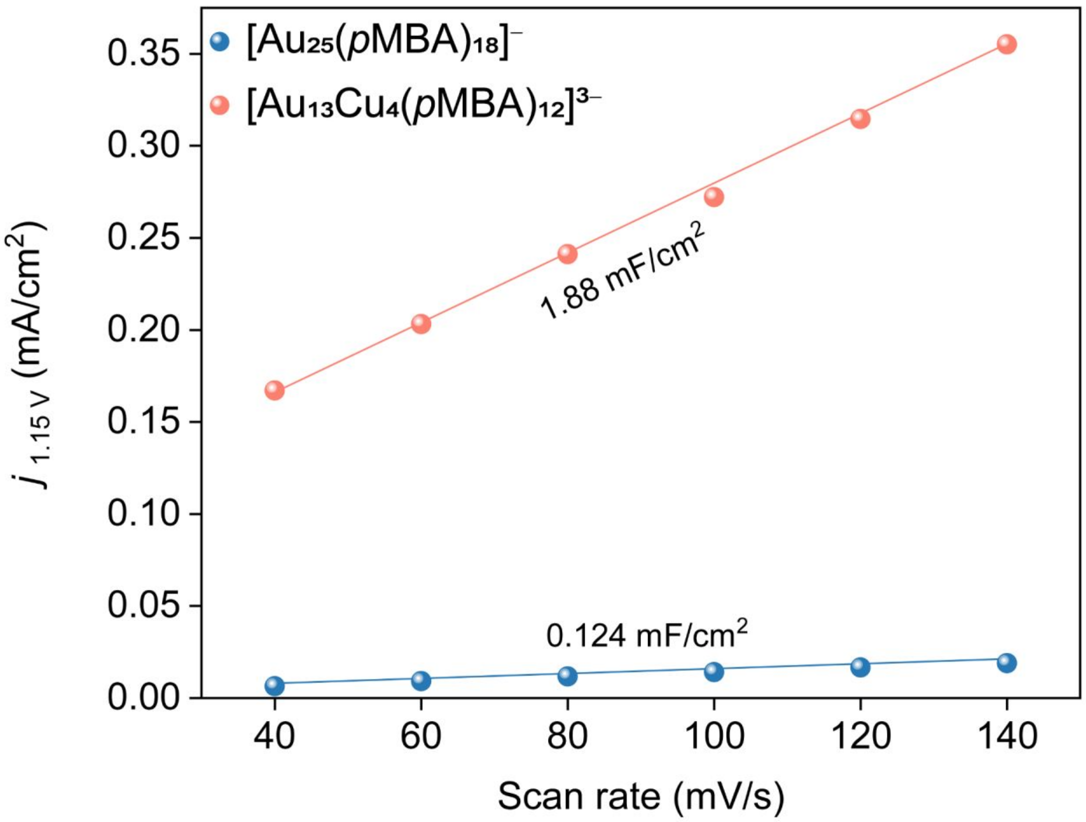

Figure S41. Electrochemical active surface area derived from double layer capacitance.

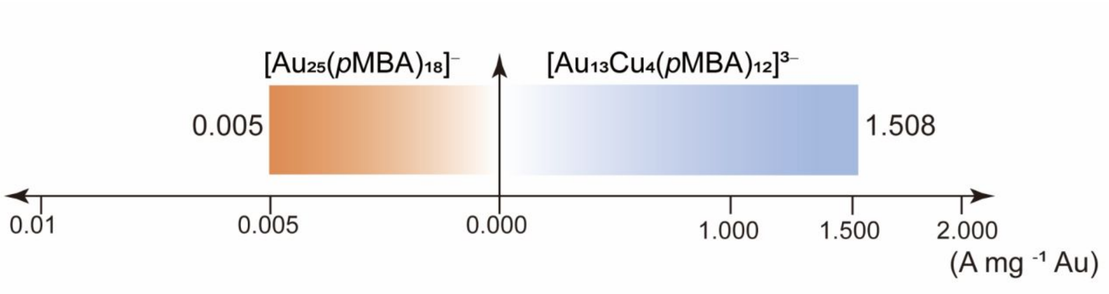

Figure S42. Mass activity at the overpotential of 200 mV.

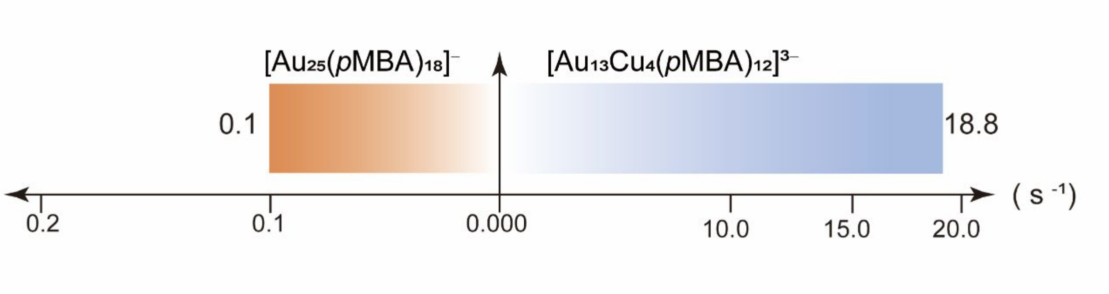

Figure S43. Turnover of frequence at the current density of 10 mA cm^{-2}.

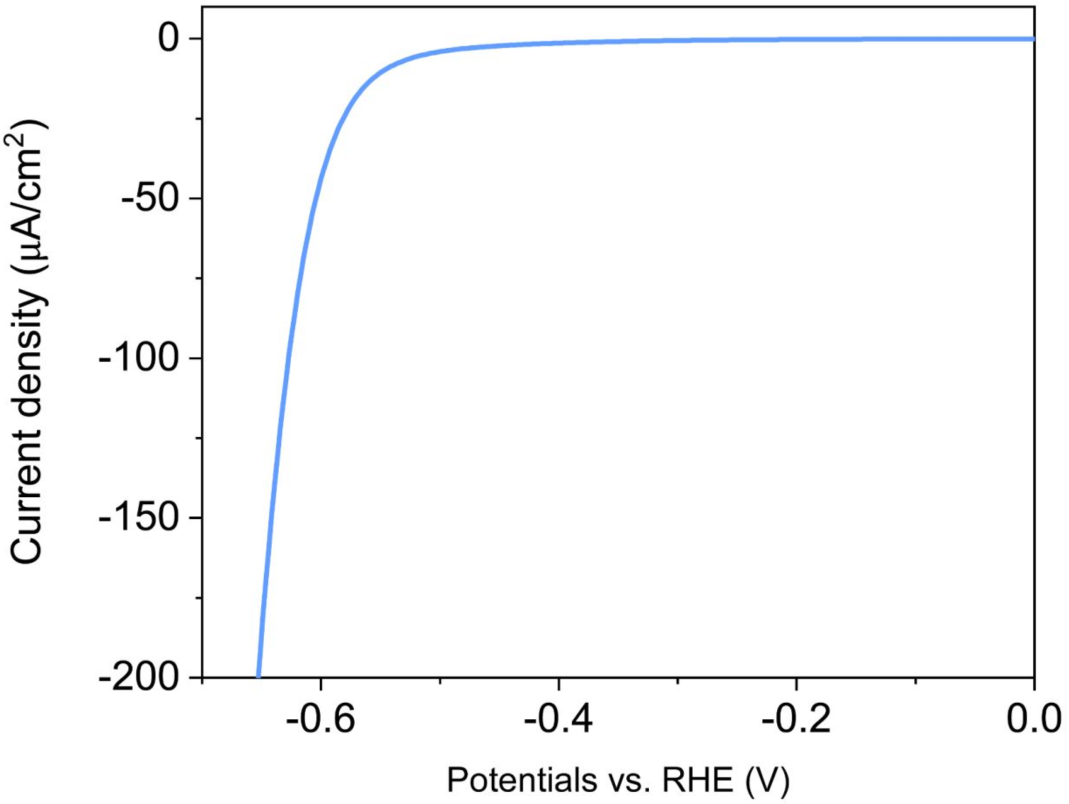

Figure S44. The LSV curve of $[Au_{13}Cu_4(p\text{MBA})_{12}]^{3-}$ with the catalyst loading of 0.06 mg cm^{-2}. To minimize the signal interference from *in situ* generated gas bubbles, we lowered the catalyst loading to 0.06 mg cm^{-2}.

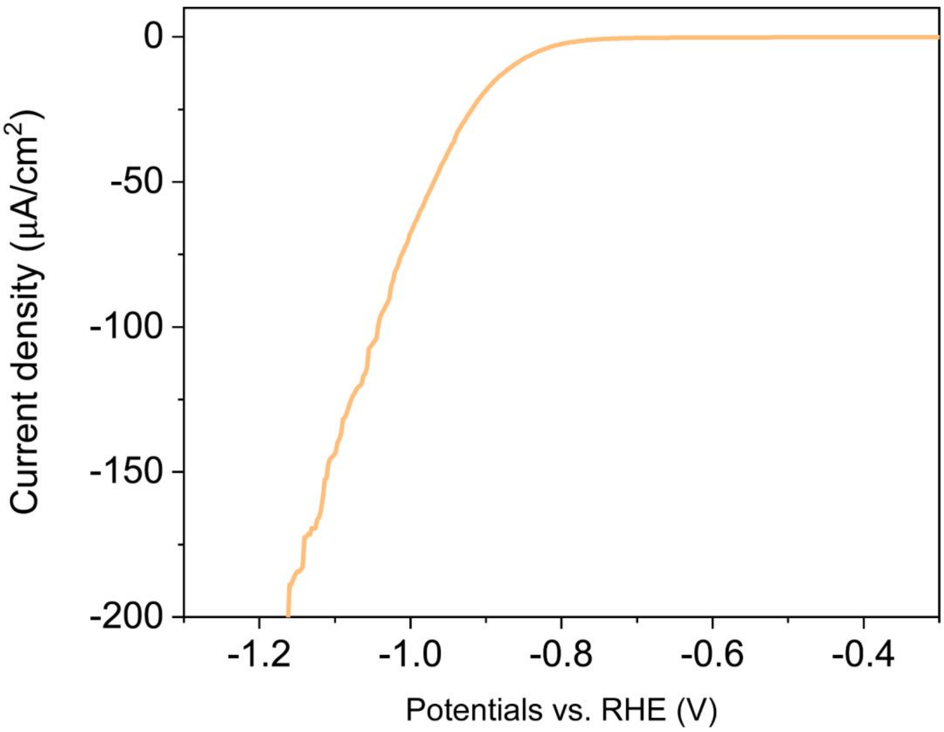

Figure S45. The LSV curve of $[Au_{25}(p\text{MBA})_{18}]^{-}$ with the catalyst loading of 0.06 mg cm^{-2}. To minimize the signal interference from *in situ* generated gas bubbles, we lowered the catalyst loading to 0.06 mg cm^{-2}.

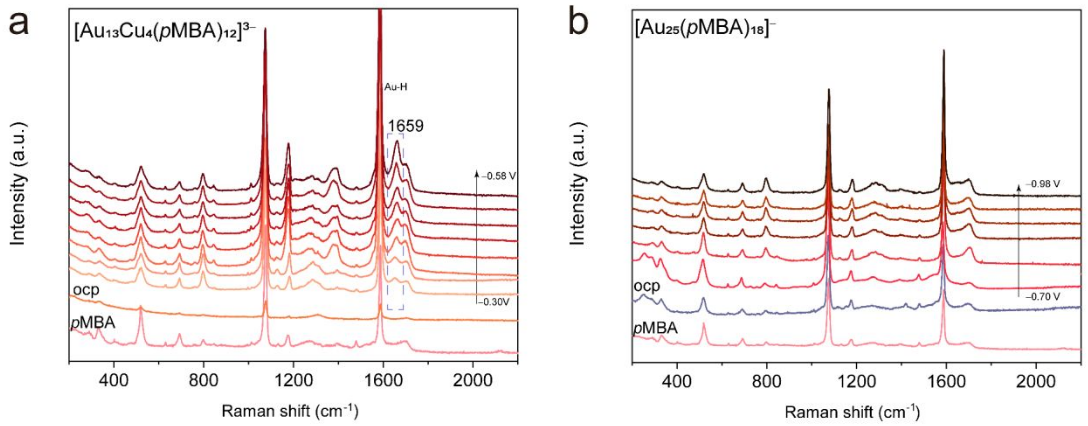

Figure S46. *In situ* Raman spectra at applied potentials. a, $[Au_{13}Cu_4(p\mathrm{MBA})_{12}]^{3-}$ NCs. b, $[Au_{25}(p\mathrm{MBA})_{18}]^{-}$.

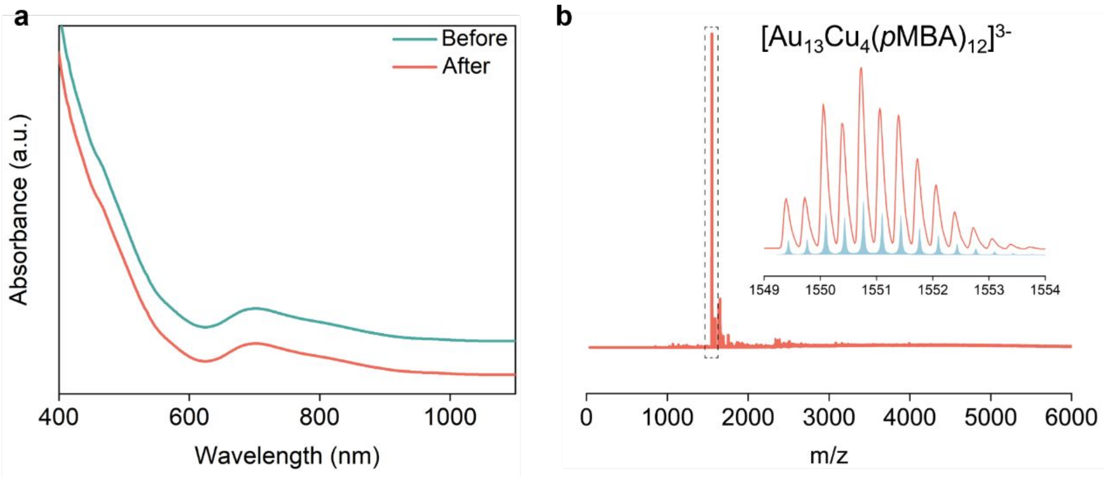

Figure S47. (a) Comparison of UV-vis absorption spectra of $[Au_{13}Cu_4(p\text{MBA})_{12}]^{3-}$ before and after HER; (b) ESI-MS of $[Au_{13}Cu_4(p\text{MBA})_{12}]^{3-}$ after HER (inset: a matched simulated isotope pattern).

Table S1 Curve fitting Parameters[a] for Au L_3 and Cu k edge EXAFS for Cu foil standard and $[Au_{13}Cu_4(p\text{MBA})_{12}]^{3-}$.

Sample	Scatting path	CN	R(Å)	σ^2 (10-3 Å)	ΔE_0	R factor
Cu foil	Cu-Cu	12	2.53±0.03	0.008±0.004	-0.15±0.46	0.003
$[Au_{13}Cu_4(p\text{MBA})_{12}]^{3-}$	Cu-S	3.3	2.23±0.02	0.009±0.003	-5.12±1.36	0.03
$[Au_{13}Cu_4(p\text{MBA})_{12}]^{3-}$	Au-S	1.6	2.28±0.01	0.007±0.001	6.93±1.31	0.027

[a] CN, coordination number; R scatting path distance; σ^2 Debye-Waller factor; S_0 was fixed as 0.9; Δ E_0 was refined as a global fit parameter(inner potential shift).

Table S2 Atom coordinates for the DFT optimized structural model for $[Au_{13}Cu_4(p\text{MBA})_{12}]^{3-}$.

Au	15.868309	15.308333	14.974167
Au	14.624140	15.502648	17.572569
Au	16.100882	17.783682	17.073581
Au	13.841653	17.242668	15.016111
Au	15.862676	12.686305	15.965410
Au	13.178666	14.583556	14.346160
Au	14.530454	16.093761	12.444744
Au	18.228173	16.907285	15.264240
Au	15.937113	13.046014	13.192339
Au	18.194209	13.624942	14.748632
Au	17.663497	15.696378	12.701051
Au	16.230965	18.009243	13.598836
Au	17.381578	15.360711	17.587996
Cu	15.959466	14.443423	10.443995
S	14.062010	15.653019	10.124524
S	15.751784	12.229019	10.946090
S	18.014383	15.365304	10.363967
C	17.339884	11.463775	10.659963
C	19.782699	10.176033	10.094995
C	18.545295	11.947914	11.202424
C	19.747063	11.309338	10.929218
C	18.579890	9.686875	9.554959
C	17.369640	10.319024	9.840042
H	18.530995	12.823182	11.849600
H	20.678174	11.676727	11.355966
H	18.597408	8.804933	8.919471
H	16.437014	9.929296	9.437027
C	12.647622	14.573394	10.157706
C	10.329588	12.974285	10.034298
C	11.546198	14.849134	10.992945
C	10.407944	14.053658	10.936081
C	11.429509	12.698191	9.201879
C	12.580427	13.482130	9.269959
H	11.597755	15.688815	11.684248
H	9.560805	14.249795	11.590423
H	11.382563	11.859339	8.511335
H	13.440037	13.252709	8.643762
C	17.919580	16.963950	9.582230
C	17.946526	19.458598	8.275302

C	17.084426	18.006933	10.020568
C	17.107039	19.241598	9.384437
C	18.757178	18.404781	7.815898
C	18.750558	17.171417	8.464245
H	16.429163	17.847431	10.875880
H	16.478679	20.056420	9.739283
H	19.405154	18.562307	6.957688
H	19.397914	16.366433	8.121781
Cu	20.276045	14.996858	16.107202
S	19.629378	14.762842	18.292442
S	20.617587	17.011617	15.191785
S	20.479066	12.946825	15.124441
C	20.962702	16.886607	13.446878
C	21.599674	16.803227	10.720129
C	21.417298	15.690715	12.866103
C	21.712987	15.645245	11.507561
C	21.143293	17.999585	11.301392
C	20.812669	18.038419	12.652526
H	21.529725	14.803044	13.484855
H	22.041819	14.720165	11.037634
H	20.432312	18.954223	13.099499
C	20.202112	16.202958	19.170354
C	21.206093	18.386614	20.636587
C	21.055895	15.992086	20.269956
C	21.558671	17.073637	20.991435
C	20.348280	18.595590	19.539934
C	19.855979	17.520182	18.813744
H	21.313405	14.974181	20.553997
H	20.079963	19.616936	19.276789
H	19.188233	17.688814	17.969967
C	20.384578	11.844906	16.519193
C	20.320217	10.130102	18.747259
C	19.279591	11.000346	16.733122
C	19.244810	10.161254	17.841583
C	21.434339	10.958506	18.520903
C	21.465218	11.810331	17.420996
H	18.442687	11.023576	16.036089
H	18.383147	9.522854	18.027175
H	22.266783	10.941261	19.219549
H	22.313026	12.473441	17.263741
Cu	15.064380	20.037203	15.071197
S	12.857219	19.466315	14.894343
S	16.515147	20.390909	13.375617

S	15.683748	20.151666	17.269104
C	18.169043	20.759762	13.913234
C	20.810470	21.416902	14.613715
C	18.677790	20.404347	15.175088
C	19.988667	20.718697	15.516058
C	20.299152	21.784272	13.355465
C	18.993523	21.450685	13.002938
H	18.045402	19.863450	15.876145
H	20.400934	20.423282	16.479297
H	20.935037	22.316109	12.652348
H	18.602336	21.712580	12.021627
C	12.324916	19.292939	13.197235
C	11.418520	18.931032	10.556536
C	11.105390	18.633031	12.948919
C	10.659472	18.454473	11.642134
C	12.628902	19.599278	10.807970
C	13.073223	19.785807	12.113722
H	10.519378	18.254685	13.784454
H	13.211657	19.962474	9.963328
H	14.007627	20.309326	12.302118
C	14.142201	20.191948	18.164198
C	11.727529	20.380045	19.604720
C	13.809658	19.220222	19.128670
C	12.610262	19.307241	19.826630
C	12.064678	21.358640	18.653081
C	13.253520	21.260306	17.933478
H	14.490794	18.390268	19.314728
H	12.336814	18.549149	20.557979
H	11.384098	22.186251	18.468863
H	13.498022	22.002054	17.176193
Cu	13.231702	12.833633	16.921918
S	12.542190	14.553328	18.269471
S	12.049042	12.605617	15.012630
S	14.867239	11.511941	17.810914
C	12.784952	11.338342	13.993770
C	13.834797	9.298678	12.372544
C	13.565358	10.314188	14.559656
C	14.091636	9.310319	13.753563
C	13.037716	10.312054	11.810622
C	12.519628	11.325263	12.612412
H	13.753599	10.310731	15.630514
H	14.713441	8.524077	14.176508
H	12.841391	10.317644	10.741763

H	11.924648	12.120999	12.169845
C	11.242396	15.422996	17.421356
C	9.069849	16.764626	16.232952
C	11.259537	16.824047	17.287438
C	10.186889	17.483850	16.695749
C	9.054063	15.363651	16.365637
C	10.127932	14.700734	16.953050
H	12.111750	17.392109	17.655129
H	10.204438	18.566206	16.583918
H	10.115553	13.616796	17.045009
C	15.426089	12.357306	19.280586
C	16.276534	13.675611	21.616441
C	14.501575	12.676980	20.292755
C	14.924142	13.330406	21.446638
C	17.202425	13.334253	20.615435
C	16.783926	12.673950	19.461431
H	13.450541	12.433948	20.153843
H	14.214332	13.601481	22.225532
H	18.250440	13.600292	20.727295
H	17.507819	12.408166	18.693325
C	10.475687	20.417844	20.389735
O	9.765540	21.594349	20.235788
O	10.077414	19.521827	21.122339
C	7.951005	17.515676	15.628824
O	6.881768	16.715020	15.280029
O	7.922224	18.725202	15.437811
C	9.088727	12.171909	10.027578
O	9.052041	11.204858	9.040740
O	8.153172	12.319798	10.802649
C	10.984376	18.763440	9.151873
O	9.753277	18.154237	9.043078
O	11.604512	19.120746	8.159245
C	16.666838	14.404143	22.844381
O	18.006208	14.719789	22.877128
O	15.916448	14.719052	23.759692
C	21.684534	19.574511	21.368190
O	22.558452	19.276452	22.398155
O	21.361319	20.728571	21.122354
C	20.230422	9.221936	19.908128
O	21.352248	9.277416	20.718803
O	19.288025	8.482298	20.162498
C	14.444653	8.216714	11.570576
O	14.078370	8.245756	10.238997

O	15.201057	7.356437	12.000346
C	21.096961	9.553810	9.834819
O	21.035682	8.482345	8.961785
O	22.161304	9.918605	10.318466
C	21.951760	16.707410	9.288183
O	22.237659	17.927271	8.719902
O	22.002854	15.672709	8.637046
C	17.945246	20.794046	7.642347
O	18.800667	20.878462	6.559523
O	17.280778	21.758376	8.001173
C	22.198233	21.721338	15.025369
O	22.879420	22.475948	14.086141
O	22.721086	21.364829	16.072524
H	8.962769	21.470119	20.778943
H	6.228472	17.327276	14.889202
H	8.196198	10.752033	9.171125
H	18.130379	15.214315	23.710444
H	22.783284	20.143850	22.787547
H	21.166927	8.645319	21.440604
H	14.545690	7.488210	9.836352
H	21.959197	8.172343	8.887464
H	22.424665	17.729551	7.781268
H	18.702638	21.795676	6.237537
H	23.771703	22.598971	14.465319
H	9.589172	18.081353	8.082750
H	8.200710	14.798083	15.999771
H	21.026949	18.887896	10.685149
H	9.722342	17.936276	11.454442
H	22.219791	16.903726	21.837620

References

(1) Liu, Z.; Chen, J.; Li, B.; Jiang, D.-e.; Wang, L.; Yao, Q.; Xie, J. Enzyme-Inspired Ligand Engineering of Gold Nanoclusters for Electrocatalytic Microenvironment Manipulation. *J. Am. Chem. Soc.* **2024,** *146*, 11773-11781

(2) Yao, Q.; Yuan, X.; Fung, V.; Yu, Y.; Leong, D. T.; Jiang, D.-e.; Xie, J. Understanding Seed-mediated Growth of Gold Nanoclusters at Molecular Level. *Nat. Commun.* **2017**, *8*, 927

(3) Kühne, T. D.; Iannuzzi, M.; Ben, M. D.; Rybkin, V. V.; Seewald, P.; Stein, F.; Laino, T.; Khaliullin, R. Z.; Schütt, O.; Schiffmann, F.; et al. CP2K: An Electronic Structure and Molecular Dynamics Software Package - Quickstep: Efficient and Accurate Electronic Structure Calculations. *J. Chem. Phys.* **2020**, *152*, 194103

(4) Hutter, J.; Iannuzzi, M.; Schiffmann, F.; VandeVondele, J. cp2k: atomistic simulations of condensed matter systems. *WIREs Computational Molecular Science* **2014**, *4*, 15-25

(5) VandeVondele, J.; Krack, M.; Mohamed, F.; Parrinello, M.; Chassaing, T.; Hutter, J. Quickstep: Fast and accurate density functional calculations using a mixed Gaussian and plane waves approach. *Comput. Phys. Commun.* **2005**, *167*, 103-128

(6) Perdew, J. P.; Burke, K.; Ernzerhof, M. Generalized Gradient Approximation Made Simple. *Phys. Rev. Lett.* **1996**, *77*, 3865-3868

(7) Grimme, S.; Antony, J.; Ehrlich, S.; Krieg, H. A Consistent and Accurate Ab Initio Parametrization of Density Functional Dispersion Correction (DFT-D) for the 94 Elements H-Pu. *J. Chem. Phys.* **2010**, *132*, 154104

(8) VandeVondele, J.; Hutter, J. Gaussian basis sets for accurate calculations on molecular systems in gas and condensed phases. *J. Chem. Phys.* **2007**, *127*, 114105

(9) Goedecker, S.; Teter, M.; Hutter, J. Separable dual-space Gaussian pseudopotentials. *Phys. Rev. B* **1996**, *54*, 1703-1710

(10) Krack, M. Pseudopotentials for H to Kr optimized for gradient-corrected exchange-correlation functionals. *Theor. Chem. Acc.* **2005**, *114*, 145-152

(11) Hartwigsen, C.; Goedecker, S.; Hutter, J. Relativistic separable dual-space Gaussian pseudopotentials from H to Rn. *Phys. Rev. B* **1998**, *58*, 3641-3662

(12) Nørskov, J. K.; Rossmeisl, J.; Logadottir, A.; Lindqvist, L.; Kitchin, J. R.; Bligaard, T.; Jónsson, H. Origin of the Overpotential for Oxygen Reduction at a Fuel-Cell Cathode. *J. Phys. Chem. B* **2004**, *108*, 17886-17892

(13) Li, J. F.; Tian, X. D.; Li, S. B.; Anema, J. R.; Yang, Z. L.; Ding, Y.; Wu, Y. F.; Zeng, Y. M.; Chen, Q. Z.; Ren, B.; et al. Surface analysis using shell-isolated nanoparticle-enhanced Raman spectroscopy. *Nature Protocols* **2013**, *8*, 52-65

(14) Ravel, B. & Newville, M. ATHENA, ARTEMIS, HEPHAESTUS: data analysis for X-ray Absorption Spectroscopy using IFEFFIT. *J. Synchrotron Radiat.* **2005**, 12, 537–541.
